# Amphioxus (*Branchiostoma floridae*) has orthologs of vertebrate odorant receptors

**DOI:** 10.1186/1471-2148-9-242

**Published:** 2009-10-05

**Authors:** Allison M Churcher, John S Taylor

**Affiliations:** 1 Department of Biology, University of Victoria, Box 3020, Station CSC, Victoria BC, V8W 3N5, Canada

## Abstract

**Background:**

A common feature of chemosensory systems is the involvement of G protein-coupled receptors (GPCRs) in the detection of environmental stimuli. Several lineages of GPCRs are involved in vertebrate olfaction, including trace amine-associated receptors, type 1 and 2 vomeronasal receptors and odorant receptors (ORs). Gene duplication and gene loss in different vertebrate lineages have lead to an enormous amount of variation in OR gene repertoire among species; some fish have fewer than 100 OR genes, while some mammals possess more than 1000. Fascinating features of the vertebrate olfactory system include allelic exclusion, where each olfactory neuron expresses only a single OR gene, and axonal guidance where neurons expressing the same receptor project axons to common glomerulae. By identifying homologous ORs in vertebrate and in non-vertebrate chordates, we hope to expose ancestral features of the chordate olfactory system that will help us to better understand the evolution of the receptors themselves and of the cellular components of the olfactory system.

**Results:**

We have identified 50 full-length and 11 partial ORs in *Branchiostoma floridae*. No ORs were identified in *Ciona intestinalis*. Phylogenetic analysis places the *B. floridae *OR genes in a monophyletic clade with the vertebrate ORs. The majority of OR genes in amphioxus are intronless and many are also tandemly arrayed in the genome. By exposing conserved amino acid motifs and testing the ability of those motifs to discriminate between ORs and non-OR GPCRs, we identified three OR-specific amino acid motifs common in cephalochordate, fish and mammalian and ORs.

**Conclusion:**

Here, we show that amphioxus has orthologs of vertebrate ORs. This conclusion demonstrates that the receptors, and perhaps other components of vertebrate olfaction, evolved at least 550 million years ago. We have also identified highly conserved amino acid motifs that may be important for maintaining receptor conformation or regulating receptor activity. We anticipate that the identification of vertebrate OR orthologs in amphioxus will lead to an improved understanding of OR gene family evolution, OR gene function, and the mechanisms that control cell-specific expression, axonal guidance, signal transduction and signal integration.

## Background

Genes encoding odorant receptors (ORs) were first identified by Linda Buck and Richard Axel in 1991 [1]. Prior to 1991, experiments from several other labs suggested that odorant receptors were seven transmembrane (TM) domain G protein-coupled receptors (GPCRs), so Buck and Axel used PCR with degenerate primers designed from available GPCR sequences to query cDNA isolated from rat olfactory epithelium tissue. The new genes they discovered were then used as probes to search rat cDNA and genomic DNA for additional paralogs [1]. This similarity-based approach, in which query sequences are used to identify orthologs and then paralogs, is a staple of both molecular and bioinformatics research. These and subsequent studies have now uncovered over a thousand rat and mouse odorant receptors [2-5] and have led to the identification of other GPCR families involved in vertebrate olfaction such as the trace amine-associated receptors (TAARs) [6], the type 1 [7] and type 2 vomeronasal receptors [8-10] and the formyl peptide receptor-like proteins [11].

In mammals, phylogenetic analyses have shown that many of the OR-encoding genes are the products of relatively recent duplication events. There are fewer OR genes in fishes, however the fish genes are more variable at the sequence level [12,13]. Despite lineage-specific gene amplification and loss, ORs in vertebrates are members of a single large monophyletic clade. Here we report the results of our search for orthologs of vertebrate ORs in the tunicate, *Ciona intestinalis *(subphylum Urochordata), and in amphioxus, *Branchiostoma floridae *(subphylum Cephalochordata).

Recently, phylogenetic analyses have shown that Urochordata is the extant sister of the vertebrates and that Cephalochordata is the sister group to the vertebrate plus urochordate clade [14], which is called Olfactores [15]. Whole genome sequences are available for *C. intestinalis *and *B. floridae*, but similarity-based surveys have not yet identified orthologs of vertebrate ORs in either genome [16,17]. However, neither study employed the available diversity of vertebrate OR sequences as queries in their survey. Here we used a bioinformatics approach that mimics the molecular strategy of Buck and Axel. Instead of degenerate primers, we used an HMM model based upon a broad diversity of full-length fish OR sequences as a probe to survey the *C. intestinalis *and *B. floridae *protein predictions. The candidate ORs identified were then used as Blastp query sequences to search within each species for additional ORs. This experiment uncovered a family of 61 OR genes in *B. floridae *but no ORs in *C. intestinalis*. Phylogenetic analyses demonstrate that the amphioxus genes we uncovered are orthologs of vertebrate ORs. Many of these new *B. floridae *sequences lack introns and are linked as is the case for most vertebrate ORs.

We identified amino acid motifs that can discriminate between ORs and non-OR GPCRs in a regular expression-based survey. These key residues may prove to be useful for identifying formerly unrecognized ORs in vertebrates and for identifying orthologs in even more distantly related taxa, such as echinoderms and hemichordates. Our results provide the foundation for future comparative studies with cephalochordates, urochordates and early vertebrates. The results will also aid in the understanding of OR gene family evolution, OR function, the mechanisms that control single receptor expression, axonal guidance, signal transduction and signal integration.

## Results

### HMM and Blastp

When we searched the *B. floridae *protein predictions using the HMM model derived from fish odorant receptors with an e-value cut off of E-10, three *B. floridae *proteins were identified. No proteins in the *C. intestinalis *protein predictions database were identified using the same search criteria. Each of the three amphioxus sequences was used as a query in a Blastp search of the *B. floridae *protein predictions. This Blastp search identified 50 sequences that were at least 40% identical to one or more of the three query sequences over a minimum of 100 amino acids. To uncover additional candidate ORs, a second Blastp search was carried out using the 50 hits from the first search as query sequences. The HMM search combined with two Blastp searches generated a list of 246 candidate ORs from the *B. floridae *protein predictions. However, 2 of the 50 hits from the first Blastp search (Braf1_106555 and Braf1_92691) had unusually long N termini and these domains alone aligned to 180 of the genes in the second Blastp search. Five more sequences were hits only to the C termini of query sequences Braf1_111311, Braf1_69444 and Braf1_87794. None of these 185 hits to N or C termini contain any of the transmembrane spanning domains and they were removed from the dataset, leaving 61 candidate amphioxus ORs (see Additional file 1). Three of these 61 proteins were previously identified as G protein-coupled receptors [16], but they were not considered to be ORs. One was classified as a basal member of the *Rhodopsin *amine family (Braf1_69014), and the other two were not classified (Braf1_109264 and Braf1_69037). Of the 61 genes, 50 are considered full-length genes because they contain all seven TM domains; the remaining 11 are partial sequences because they are missing at least one of the seven TM domains.

### Phylogenetics

**Figure 1:**
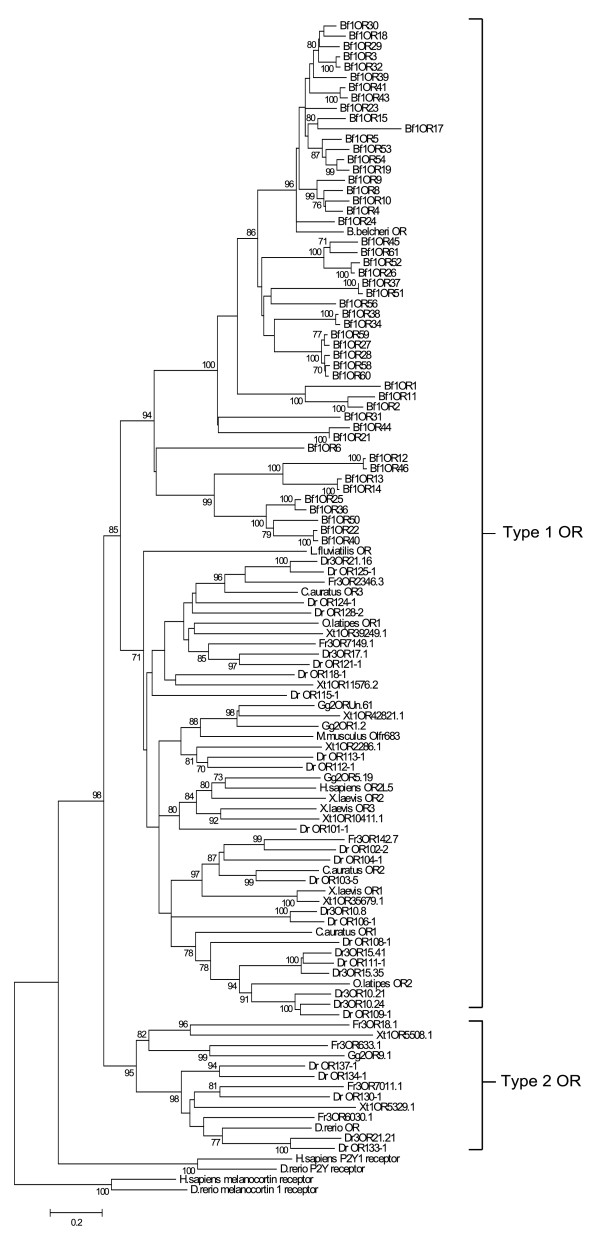
**Phylogenetic analysis of *B. floridae *and vertebrate type 1 and type 2 ORs**. Neighbor-Joining tree of representative type 1 and type 2 vertebrate ORs (n = 59), 50 full-length *B. floridae *ORs, and one sequence from *B. belcheri *[22]. The vertebrate ORs include sequences from human, mouse, fish, amphibian, chicken and lamprey. The abbreviations in the sequence names represent these species: *Danio rerio *(Dr), *Fugu rubripes *(Fr), *Xenopus tropicalis *(Xt), and *Gallus gallus *(Gg). Type 1 and type 2 designations are based on [12]. Tree construction was based on approximately 200 amino acid positions and 1000 bootstrap replicates were conducted. The bootstrap values for nodes with less than 70% support were excluded from the tree and human and fish purinergic [GenBank:NM_002563, GenBank:CAK04925] and melanocortin receptors [GenBank:AAC13541, GenBank:NP_851301] were used to root the tree. For the complete list of vertebrate ORs included in the tree, see Additional file 2.

We aligned the 50 full-length candidate ORs from *B. floridae *with vertebrate ORs (see Additional file 2 for sequence list), some of which were used in the construction of the HMM. We also included non-OR GPCRs from the *Rhodopsin *family to root the tree (alignment shown Additional file 3). The OR and non-OR out-group sequences have several 'anchor' residues common to *Rhodopsin *family GPCRs. These features include: a conserved cysteine residue in transmembrane domain three, TM3 [18,19], the conserved E/DRY motif at the junction of TM3 and intracellular loop two (IL2), a tryptophan residue in TM4, and the NPxxY motif in TM7 [20,21]. These conserved sites were used to obtain a reliable alignment. The results of this analysis (Figure 1) suggest that *B. floridae *ORs fall into two subfamilies: one contains 40 genes, the other contains 10 genes. The phylogeny also shows that all *B. floridae *candidate ORs belong to a monophyletic clade and that this clade is the sister group to type 1 vertebrate ORs. This last observation suggests that vertebrate type 2 ORs diverged from type 1 ORs prior to the split between cephalochordates and the Olfactores [15]. Finally, a single gene from *Branchiostoma belcheri*, believed to be an amphioxus OR based on its expression domain [22], occurs in the larger subfamily of *B. floridae *ORs.

### Regular expression survey

**Figure 2:**
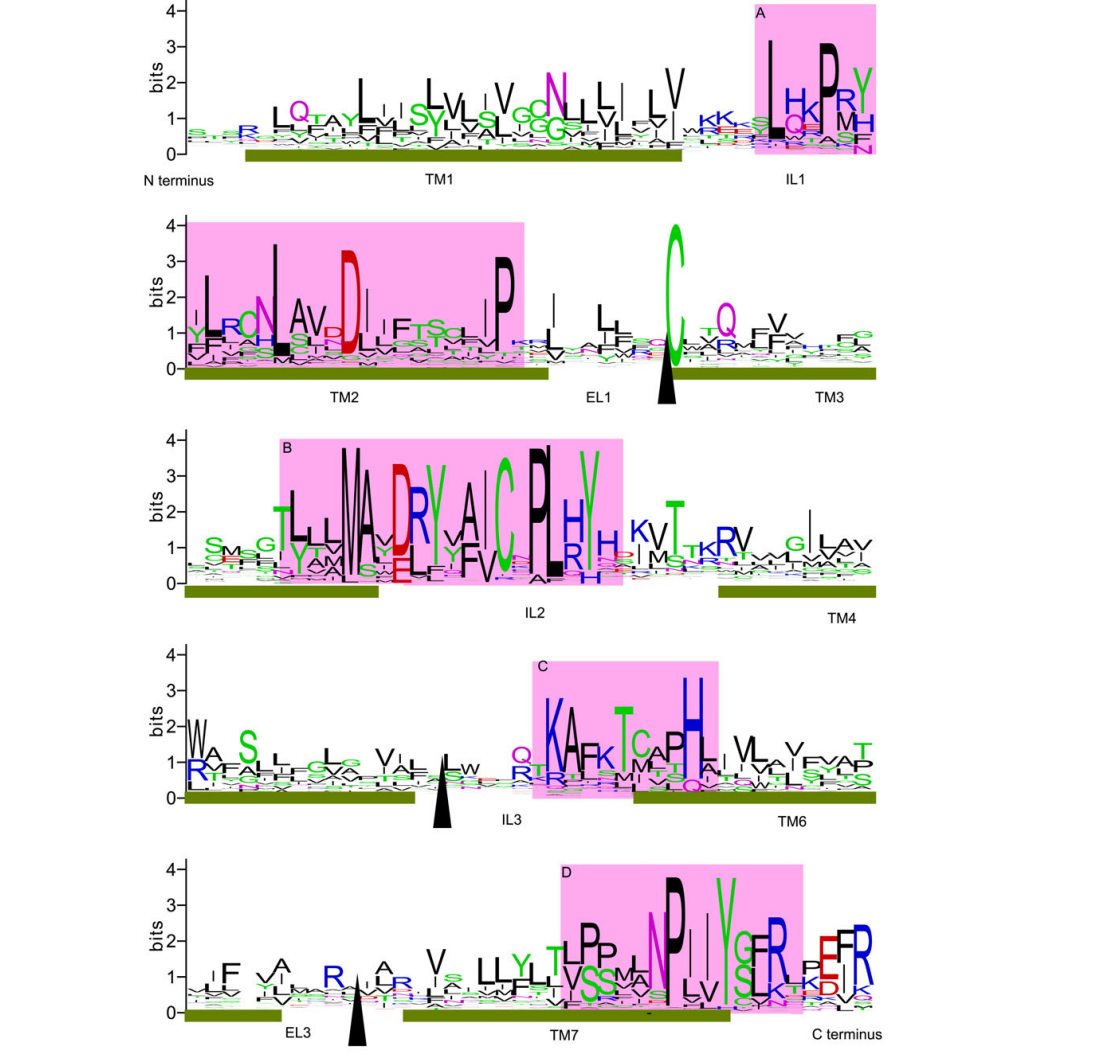
**WebLogo based on an alignment of type 1 and type 2 vertebrate ORs and *B. floridae *ORs**. This WebLogo was created using an alignment of 64 vertebrate ORs (chicken, human, mouse, lamprey, amphibian and fish), 50 full-length *B. floridae *ORs and 11 partial *B. floridae *ORs. Approximately 200 positions were included in the alignment. The arrows indicate positions where blocks of sequence have been removed because they could not be aligned. For this reason, extracellular loop 2 (EL2) and transmembrane domain 5 (TM5) are missing from the WebLogo image. Transmembrane domains are represented by green bars and the boundaries were defined according to [45]. The intracellular and extracellular loops are labelled 'IL' and 'EL' respectively. The four conserved regions shaded in pink (labelled A-D) were used to generate a list of OR motifs (see Table 1).

The phylogenetic node separating amphioxus and Olfactores [15] is approximately 550 million years old [23]. By identifying individual amino acid residues or motifs that are conserved in amphioxus and vertebrate ORs we may be able to find those that play an important role in OR function. Four conserved regions were uncovered using WebLogo (Figure 2). Three of these are found in intracellular loops 1-3, and one is found in TM7. For each of these conserved regions, it was possible to derive between 1 and 12 sub-motifs that could be evaluated in terms of their ability to discriminate between ORs and other GPCRs from the *Rhodopsin *gene family. These motifs were used in regular expression searches of an OR and a non-OR MySQL databases. From this list we identified one motif (KAxxTxxxH) that is found in more than 73% of ORs and less than 1% of non-ORs, and two motifs (MxxxxYxxxCxPLxY, and LxxPxYxxxxxLxxxDxxxxxxxxP) that are present in more than 44% of ORs and less than 1% of non-ORs (Table 1).

**Table 1:** List of amino acid motifs used to search OR and non-OR sequence databases

	**Motif**	**ORs**	***B. floridae *ORs**	**Non-ORs**
A	L..P.Y.L...L...D........P	5.79	4.92	0.04
A	L..P.Y.....L...D........P	44.43	8.20	0.06
A	L..P.......L...D.........P	47.11	77.05	7.56
A	L..P...L...L...D........P	6.71	73.77	5.39
B	L...M....Y...C.PL.Y	32.93	0.00	0.00
B	L...MA.D.Y...C.PL.Y	25.27	0.00	0.00
B	M....Y...C.PL.Y	52.94	67.21	0.17
B	MA.D.Y.AIC.PL.Y	37.57	0.00	0.01
B	MA.D.Y...C.PL.Y	41.69	37.70	0.10
B	MA.DRY.AIC.PL.Y	36.02	0.00	0.01
B	MA...Y...C.PL.Y	42.04	63.93	0.16
C	KA..T...H	73.48	14.75	0.24
C	KAF.....H	50.24	42.62	0.17
C	KA......H	75.05	47.54	1.54
C	K...T...H	83.32	18.03	1.29
D	NP..Y	68.90	75.41	40.03
D	NP..Y..R	46.41	65.57	2.45
D	NP..YG	7.87	21.31	6.17
D	NP..YS	36.43	54.10	2.11
D	NPIIY	13.63	42.62	9.74
D	P..NP..Y	57.32	0.00	0.59
D	PP..NP..Y	15.45	0.00	0.02
D	PS..NP..Y	0.28	3.28	0.01
D	S..NP..Y	0.97	65.57	21.65
D	S...NP..Y	2.57	62.30	8.36
D	SP..NP..Y	1.86	0.00	0.05
D	SS..NP..Y	0.15	62.30	2.77

This table contains the 27 amino acid motifs identified using the WebLogo of *B. floridae *and vertebrate ORs. The areas of conservation from which the motifs were derived are labelled A-D and correspond to the regions shaded in pink in Figure 2. All variable amino acid positions are denoted by periods. The OR motifs were used in regular expression searches of an OR sequence database (n = 5438), a non-OR database (n = 21 282) and a database of full-length and partial *B. floridae *ORs (n = 61). The occurrence of each motif in a given database is given as a percentage.

### Amphioxus OR gene structure and location

Of the 61 *B. floridae *ORs, 50 are considered to be full-length genes. Of these, 35 are predicted to be intronless (see Additional file 1). In the *B. floridae *assembly version 2.0, the 61 genes are found on 44 scaffolds. Four of these scaffolds contain two ORs, two contain three ORs and three contain four ORs.

## Discussion

Using a combination of HMM and Blastp searches, we have identified 50 full-length and 11 partial sequences among the *B. floridae *protein predictions that appear to be odorant receptors (ORs). Similarities between the vertebrate ORs used to generate the HMM and amphioxus hits to this HMM are low. However, the stringent criteria used in our alignment-based searches and the bootstrap support for the key nodes in the phylogenetic tree support the hypothesis that these amphioxus genes are orthologs of vertebrate odorant receptors. Furthermore, the *B. floridae *candidate ORs have amino acid motifs found in vertebrate ORs that appear not to occur, or occur very rarely, in non-OR genes from the *Rhodopsin *family. Lastly, evidence has been reported (see below) indicating that these genes are likely to be expressed in *B. floridae *rostral epithelium.

### Phylogenetic analysis

Vertebrate ORs have recently been divided into two groups, the type 1 and the type 2 ORs; the type 1 genes have been further subdivided into six clades [12]. Genes from only two of these type 1 clades are present in mammals, whereas fish and amphibians have genes from five of the six clades. Type 2 ORs have been subdivided into three clades and appear to be present only in amphibians and fish [12]. Since type 1 ORs have been identified in lamprey [24], the divergence between these two lineages of paralogous genes occurred at least 450 million years ago [12]. Representatives from all nine type 1 and type 2 vertebrate OR clades were included in a phylogenetic analysis with the candidate ORs from *B. floridae *identified here. The results of this analysis demonstrate that amphioxus ORs and the vertebrate type 1 ORs form a monophyletic group (Figure 1). In a separate phylogenetic analysis, we added fish and mammalian sequences from the α, β, γ and δ groups of *Rhodopsin *GPCRs [20] and non-OR *Rhodopsin*-like GPCRs from *B.floridae *[16] (see Additional file 4). The addition of more sequences to the phylogeny had no effect on the bootstrap support for the key nodes and did not change the topology of the tree. These observations not only provide strong support for the hypothesis that the amphioxus genes are orthologs of vertebrate ORs, they also indicate that type 1 and type 2 ORs diverged more than 550 million years ago.

Sequence identity among amphioxus ORs ranges from approximately 22% to 95%, over the seven transmembrane regions indicating that these genes were produced by old and recent duplication events. This pattern can also be observed in fish ORs; sequence identity among the 238 fish ORs used in this study ranges from under 20% to over 90% (data not shown). The range of sequence identity values between *B. floridae *ORs and the vertebrate ORs derived from the alignment used to reconstruct the phylogeny in Figure 1 was 10% to 31%. All *B. floridae *ORs are members of a clade that contains no vertebrate sequences suggesting that a few OR genes have provided the raw material for gene family expansions just as in several vertebrate lineages.

The number of OR genes identified in *B. floridae *is smaller than the number of OR genes that are found in most vertebrates. One possible explanation is that the majority of the receptors involved in olfaction in *B. florida*e are encoded by other gene families, such as the TAARs or the formyl peptide receptor-like proteins. Alternatively, we may not have identified all members of the *B. floridae *OR gene family. If this is the case, these genes may belong to OR gene families yet to be identified in any chordates; the InterPro database [25] contains a number of orphan GPCRs in the *Rhodopsin *family. As genome annotation improves for lamprey, hemichordates and echinoderms, it might be possible to identify additional OR genes in amphioxus and vertebrates that cannot be detected using a search based entirely upon the OR diversity currently described in vertebrates.

As mentioned above, Nordström et al. [16] did not uncover orthologs of vertebrate ORs among the 664 GPCRs identified in their survey of the *B. floridae *protein predictions. Our search strategy differed from theirs in that it employed a greater diversity of vertebrate OR sequences to query the amphioxus protein predictions. As mentioned above, mammals generally have more ORs than fish, but they have representatives of only two of the nine OR clades, whereas fish have OR genes from eight of these clades [12]. By using fish sequences instead of mammalian sequences in our search, we emphasized residues conserved in a broad diversity of ORs and were able to ignore residues that appear to be diagnostic for ORs only because they are common in recently duplicated genes.

### Sequence conservation: GPCRs

The candidate *B. floridae *ORs identified in this study share several features with other genes in the *Rhodopsin *family of GPCRs. These include a conserved cysteine residue at the border of TM3 and extracellular loop (EL1), a conserved tryptophan in TM4, and an NPxxY motif (where x represents a variable amino acid position) in TM7. The cysteine residue is present in most GPCRs and is thought to participate in a disulfide bond between TM3 and EL2 [18,19]. The tryptophan residue in TM4 plays a role in inter-helix interactions that help to maintain receptor conformation [26]. The NPxxY motif is found in most GPCRs in the *Rhodopsin *family [18,20] including vertebrate ORs [13,27] and is thought to be involved in receptor internalization and desensitization [28]. A DRY motif occurs in TM3 of most *Rhodopsin *family GPCRs [18,20]. While this motif is also present in some *B. floridae *ORs, the majority have a leucine (L) in place of the arginine (R) residue. The consequences of mutations in this motif vary [reviewed in [29]] and the DLY motif is not inconsistent with OR status. A search of our InterPro OR database uncovered homologous DLY motifs in human, colobus monkey, and dolphin OR proteins.

### Sequence conservation: odorant receptors

Having shown that *B. floridae *ORs share several sequence features with other members of the *Rhodopsin *family of GPCRs, our next goal was to identify features specific to ORs. The WebLogo analysis of an alignment of 125 ORs revealed four areas that are conserved in vertebrate and amphioxus ORs (Figure 2). From these four regions, we generated a series of 27 motifs which were then tested for their ability to discriminate between ORs and non-ORs. This survey identified three motifs common in ORs but rare in other *Rhodopsin *family GPCRs; LxxPxYxxxxxLxxxDxxxxxxxxP, MxxxxYxxxCxPLxY and KAxxTxxxH. These three motifs are found in intracellular loops one, two and three respectively and all three overlap with neighbouring TM domains. The KAxxTxxxH was best at discriminating between ORs and non-ORs (as defined by InterPro). This motif occurred in 73.48% of ORs, but only in 0.24% of non-ORs.

Conserved amino acid motifs have previously been noted in alignments of human, mouse and zebrafish OR sequences [13,27,30,31] and these motifs include some of the amino acid residues highlighted above. For example, most mammalian ORs have a conserved motif in IL1 [31] that is similar to the first motif identified in this study. Both motifs include a leucine (L) residue followed by downstream proline (P) and tyrosine (Y) residues. In *B. floridae *ORs, the L, P and Y residues are conserved though the Y residue appears to have been lost in many of the recent duplicates. Also, most human odorant receptors have the MAYDRYVAIC motif at the border of TM3 and IL2 [27] and this motif can also be found in mouse [4] and zebrafish ORs [13]. The comparison between the MAYDRYVAIC motif and the second motif identified here suggests that the methionine (M), tyrosine (Y), and cysteine (C) residues are the most important components of this motif and may have OR-specific functions. The alanine (A) and aspartic acid (D) residues are also common in both vertebrate and *B. floridae *ORs (Table 1). In IL3, the KAFSTC motif is also present in human, mouse and zebrafish ORs [4,13,27], however, the phenylalanine (F) and serine (S) residues are not as common in zebrafish ORs. The comparison between the KAFSTC motif and our third motif suggests that the lysine (K), alanine (A) and threonine (T) residues play the most important roles, and that the downstream histidine (H) would also be a good candidate for site directed mutagenesis studies. Though the threonine residue is highly conserved between taxa, it appears to have been lost in many of the *B. floridae *ORs. Finally, our analysis that included *B. floridae *sequences shows that an NPxxY motif, which is common to *Rhodopsin *family GPCRs, becomes a good OR marker when an arginine (R) residue is included two amino acids positions downstream (i.e. NPxxYxxR).

The locations of the motifs within the intracellular loops suggest they these loops are important for OR signalling. In other GPCRs, the intracellular loops interact with G proteins and other proteins on the inside of the cell to regulate signal transduction. In mOR-EG, a mouse OR, mutation of conserved positions within the intracellular loops has been shown to inhibit receptor function that is unrelated to the protein's ability to bind ligands [32]. The pattern of conservation observed here suggests that signal transduction in both cephalochordate and vertebrate sensory neurons may be regulated by similar molecular interactions on the inside of the cell. These conserved residues may also be important for maintaining receptor conformation in cephalochordates and vertebrates. Though purely speculative as to what the precise role of these residues is, these sites, because of their persistence over evolutionary time, are excellent candidates for functional analysis.

### Organization in genome

ORs in vertebrates are intronless and have short N and C termini [1,2,4]. In *B. floridae*, 35 out of 50 of the full-length ORs identified in this study are intronless. Like vertebrates, most *B. floridae *ORs have short N termini but unlike vertebrates, many *B. floridae *ORs have long C termini. In mOR-EG, the C terminus plays an important role in maintaining receptor conformation and specificity; mutation of residues within the C terminus can inhibit signalling [33]. In other GPCRs, the C terminus is important for receptor phosphorylation and the internalization of the receptor from the membrane [reviewed in [34]]. The presence of long C termini in *B. floridae *ORs should be confirmed experimentally, however, the presence of several clusters of serine and threonine residues in the C termini suggests they may be sites for receptor phosphorylation as seen in other GPCRs [34,35].

Another common feature of vertebrate odorant receptors is that they are often found tandemly arrayed in the genome [2,4,13,36,37]. More than half of the full-length and partial OR genes identified here are found on a scaffold with at least one other OR, and over a third of these genes are found on a scaffold with two or more ORs. Since the *B. floridae *genome assembly is not yet complete, the degree of linkage between *B. floridae *ORs is likely an underestimate.

### Expression in the rostral epithelium

Although our bioinformatics approach tells us only that these amphioxus genes are orthologs of vertebrate ORs, there are also experimental data for a similar gene in *B. belcheri *suggesting these genes function as ORs. Satoh [22] sequenced a single gene from *B. belcheri *that appeared to be related to vertebrate ORs and he showed expression in the rostral epithelium using an in-situ probe for this sequence. This gene was included in our phylogenetic analysis of *B. floridae *candidate ORs and it occurred nested within the larger group of *B. floridae *ORs (Figure 1). Interestingly, Satoh also mentioned that the sequence he amplified from cDNA for the in-situ probe was 'nearly identical' to the one derived from genomic DNA suggesting there may be recently duplicated OR genes in the *B. belcheri *genome that are highly similar in primary sequence. If these duplicate genes are present in the *B. belcheri *genome as seen in the *B. floridae *genome, then the primers used to make the in-situ probe may not have been gene-specific resulting in a pool of probes generated from highly similar *B. belcheri *OR genes. Alternatively, a single probe may have bound to multiple, highly similar mRNAs. These factors may explain the 'ubiquitous' expression pattern in the rostral epithelium that Satoh observed.

In conjunction with the expression data collected by Satoh [22], the identification of amino acid motifs that are conserved in both amphioxus and vertebrate ORs supports the hypothesis that these amphioxus genes function as ORs. However, GPCRs that are similar in sequence may not have exactly the same function: sequence identities among the formyl peptide receptor-like genes range from 67-96% but in mice, not all of these genes are expressed in the vomeronasal sensory neurons [11]. For this reason, further experimental evidence is required to determine if the amphioxus ORs have the same function as vertebrate ORs.

### *Ciona intestinalis*

Using the search strategy employed here, we did not uncover orthologs of vertebrate ORs in the urchordate *Ciona intestinalis*. Our results are consistent with those obtained in a recent survey of the *C. intestinalis *protein predictions for GPCRs [17]. Orthologs of vertebrate ORs may be present in other urochordate species but have been lost in *C. intestinalis*. However, the results of our phylogenetic analysis show that OR families have expanded from a few progenitor genes independently in many lineages, suggesting that the loss of ORs in any one clade (e.g. urochordates) could have been be a result of the loss of only one or two ancestral genes.

## Conclusion

In this study we have identified orthologs of vertebrate odorant receptor genes in the cephalochordate *B. floridae*. This discovery supports the hypothesis that vertebrate odorant receptors evolved prior to the split between cephalordates and chordates which occurred approximately 550 million years ago [23]. By aligning and comparing vertebrate and amphioxus ORs, we have identified amino acid motifs that are conserved only in ORs. These residues may prove useful for uncovering formerly unrecognized ORs in vertebrates and for uncovering orthologs in more distantly related taxa. These sites, which occur in intracellular loops, are also excellent candidates for mutation-based study of OR signal transduction. The expression domains of these genes may be used to identify homologous sensory neurons in vertebrate and invertebrate chordates. Comparative studies that include cephalochordates, urochordates and early vertebrates should help us to understand OR gene family evolution, the mechanisms that control single receptor expression, axonal guidance, and signal transduction and integration.

## Methods

### An HMM and Blastp based search for odorant receptors in *B. floridae*

Ray-finned fish (Actinopterygii) odorant receptors (n = 238), the majority from zebrafish (*Danio rerio*), were used to create an odorant receptor (OR) Hidden Markov Model (HMM). We used fish sequences instead of mammalian ORs because fishes have retained members of eight of the nine classes of odorant receptors thought to be present in early vertebrates [12]. Although mammals possess on average more ORs than other vertebrates, only two of the nine OR clades are present in mammals [12]. Fish OR sequences [13] were downloaded from GenBank and translated into proteins. All pseudogenes, and one sequence that could not be aligned (NM_131143.1), were removed and the remaining sequences were aligned with ClustalW [38]. The alignment was edited using BioEdit [39] and used to construct a profile hidden Markov model (HMM) using default settings and the HMM calibrate application [40,41]. This HMM model was used to search the *B. floridae *protein predictions (N = 50 817, assembly v1.0) that were downloaded from the DOE Joint Genome Institute [42]. The protein predictions for *Ciona intestinalis *(N = 19 858, assembly version 2 release 53) were downloaded from Ensemble [43]. An E-value cut-off of E-10 and default parameters were used for the HMM searches.

The *B. floridae *sequences identified in the HMM survey were used as query sequences in a Blastp [44] search of the *B. floridae *protein predictions. For a Blastp hit to be considered a candidate OR, it had to be at least 40% identical to the query sequence over a minimum of 100 amino acids. Each of the hit sequences that met this criterion was used in a second Blastp search using the same criteria. In this survey, only hits to at least part of any of the TM-spanning domains of the query sequence were retained. Sequences that spanned all seven TM domains were considered full-length sequences; all others were considered partial sequences.

### Phylogenetic analyses

All candidate ORs from *B. floridae *were aligned to 59 vertebrate ORs including sequences from lamprey, tetrapod (Sarcopterygii), and ray-finned fish (Actinopterygii) using ClustalW [38]. A single candidate OR from *B. belcheri *[22] was also included in the alignment. N and C termini were removed for phylogenetic analysis as well as EL2 and TM5 because they could not be aligned (transmembrane boundaries defined by Man et al. [45]). Non-OR GPCRs from the *Rhodopsin *family included in the alignment were human and fish purinergic [GenBank:NM_002563, GenBank:CAK04925] and melanocortin receptors [GenBank:AAC13541, GenBank:NP_851301]. Although several other *Rhodopsin*-like genes were used as out-groups in our preliminary analyses (see Additional file 4), the P2Y and melanocortin receptors were chosen because the human P2Y receptor belongs to a subgroup of the *Rhodopsin*-like GPCRs that includes the human ORs (group δ) and is expected to be more closely related to the vertebrate ORs than the melanocortin receptor which belongs in another subgroup (group α) [20]. An alignment of 200 amino acid positions was used to construct a Neighbor-Joining tree in Mega3.1 [46] based on Poisson-corrected distances. Support for tree topology was estimated using 1000 bootstrap replicates.

### Key amino acid motifs

To identify amino acid motifs common in vertebrate and *B. floridae *ORs, we constructed an alignment of vertebrate (n = 64) and *B. floridae *(n = 61) ORs. Sequences from all nine clades of vertebrate ORs [12] were used in the alignment including representatives from human, mouse, lamprey, fish, chicken and amphibians. The alignment included the same amino acid positions that were used for the phylogeny. Using the alignment, we constructed a WebLogo [47] from which a list of candidate amino acid motifs was generated. To determine whether these motifs were present in ORs, non-ORs, or both, we downloaded InterPro protein families [25] IPR000276 (*Rhodopsin*-like GPCRs) and IPR000725 (Olfactory receptors) and used them to construct two MySQL databases: one containing 5438 odorant receptors and the other containing the *Rhodopsin*-like sequences with the OR genes from IPR000725 excluded (N = 21 282). We searched these databases for the presence of the motifs using a series of regular expressions. An OR-specific motif was defined as one that is found in a large proportion of ORs but less than 1% of non-ORs.

### OR gene structure and scaffold positions

Vertebrate odorant receptors are intronless and are often found in tandem [1,48,49]. To determine if *B. floridae *ORs are also intronless and in tandem, we obtained exon number and gene orientation from the annotation file accompanying genome assembly v1.0. The locations of these genes were obtained from the more recent version of the assembly, v2.0. Our ability to identify single exon genes is limited by the incomplete annotation of the genome. However, as previously stated, we considered a full-length sequence to be one that spans all seven transmembrane domains.

## Authors' contributions

AMC and JST conceived and designed this study. AMC collected and analyzed the data and JST assisted with sequence alignments. Both authors contributed to the writing of the manuscript and have read and approved the final manuscript.

## Supplementary Material

Additional file 1 **List of full-length and partial *B. floridae *ORs**. This file contains *B. floridae *protein IDs, intron and exon information, and the location of each gene in the *B. floridae *genome assembly (v2.0). Full-length and partial sequences are denoted by 'F' and 'P' respectively. Click here for file

Additional file 2 **List of vertebrate odorant receptors and non-OR GPCRs used in the phylogenetic analyses**. This file contains a list of sequences used in the phylogenetic analyses and the GenBank accession numbers where available. Click here for file

Additional file 3 **Multiple sequence alignment used for the phylogenetic analyses**. This file contains the multiple sequence alignment used to generate the Neighbor-Joining trees in Figure 1 and Additional file 4. The N and C termini have been removed as well as three blocks of sequence that could not be aligned (shown as gaps in the alignment). Click here for file

Additional file 4 **Phylogenetic analysis of vertebrate ORs, B. floridae ORs and non-OR Rhodopsin-like GPCRs**. This file contains an unrooted Neighbor-Joining tree constructed using vertebrate ORs, cephalochordate ORs and non-OR GPCRs from the α, β, γ and δ groups of GPCRs from the *Rhodopsin *family [20]. Non-OR GPCRs include melanocortin, trace amine-associated, alpha2B-adrenergic, adenosine, oxytocin, opioid, somatostatin, hypocretin, mas-related, formyl peptide and purinergic receptors. Fish-mammalian pairs of receptors were included where possible as well as non-OR *B. floridae *GPCRs identified by [16] (see Additional file 2 for the complete sequence list). Tree construction was based on approximately 200 amino acid positions and 1000 bootstrap replicates were conducted (see Additional file 3 for sequence alignment). Click here for file
