# Supplementary material for: Amphioxus (Branchiostoma floridae) has orthologs of vertebrate odorant receptors
Source: BMC Evol Biol. 2009 Oct 5;9:242. doi: 10.1186/1471-2148-9-242 (PMC2764704; doi:10.1186/1471-2148-9-242)
Supplement: Additional file 1 — List of full-length and partial B. floridae ORs. This file contains B. floridae protein IDs, intron and exon information, and the location of each gene in the B. floridae genome assembly (v2.0). Full-length and partial sequences are denoted by 'F' and 'P' respectively. [file 1471-2148-9-242-S1.PDF]

Gene name	F/P	Protein ID	Scaffold	Strand	First position	Last position	Length	Exon
Bf1OR1	F	64125	3	-	3717128	3718141	1014	1
Bf1OR2	F	64126	3	-	3721488	3722979	996	2
Bf1OR3	F	88923	17	+	914257	915669	1413	1
Bf1OR4	F	73254	21	+	3560843	3562279	1437	1
Bf1OR5	F	69014	21	-	3166004	3167407	1404	1
Bf1OR6	F	92979	28	+	2002204	2003346	1143	1
Bf1OR7	P	87794	32	-	748342	753305	1020	2
Bf1OR8	F	71292	32	+	3089890	3091416	1527	1
Bf1OR9	F	71300	32	-	3199315	3200694	1380	1
Bf1OR10	F	71301	32	-	3205527	3207134	1608	1
Bf1OR11	F	64115	41	-	2513326	2514741	960	2
Bf1OR12	F	118526	62	+	4296209	4320444	1143	16
Bf1OR13	F	67646	65	-	4287947	4289206	1260	1
Bf1OR14	F	67647	65	-	4290352	4294255	1200	2
Bf1OR15	F	77183	69	+	521986	523437	1452	1
Bf1OR16	P	78872	80	+	738618	765389	591	3
Bf1OR17	F	92603	93	-	1889505	1899289	1044	3
Bf1OR18	F	80472	94	-	42507	44096	1590	1
Bf1OR19	F	73439	98	-	4245059	4246525	1467	1
Bf1OR20	P	198258	100	+	110307	110642	336	1
Bf1OR21	F	93291	105	-	21149	22282	1134	1
Bf1OR22	F	83327	120	-	862466	863749	1284	1
Bf1OR23	F	81320	128	-	66887	68182	1296	1
Bf1OR24	F	81319	128	-	61007	62209	1203	1
Bf1OR25	F	85038	137	+	1434351	1435553	1203	1
Bf1OR26	F	77393	155	+	848952	850472	1521	1
Bf1OR27	F	80556	157	-	990066	991424	1359	1
Bf1OR28	F	223001	157	+	985356	986276	921	1
Bf1OR29	F	82317	157	+	2847848	2849272	1425	1
Bf1OR30	F	78920	157	-	2184721	2185995	1275	1
Bf1OR31	F	87788	166	-	426318	431866	1065	2
Bf1OR32	F	88953	180	-	1472087	1476371	1062	2
Bf1OR33	P	108091	192	+	80940	81950	1011	1
Bf1OR34	F	90545	199	-	184564	198429	1854	4
Bf1OR35	P	69037	200	-	1629971	1636017	1116	2
Bf1OR36	F	88114	205	-	1393258	1394559	1302	1
Bf1OR37	F	121476	216	-	1876011	1883409	2550	7
Bf1OR38	F	69444	220	+	1546261	1548350	1935	2
Bf1OR39	F	92691	226	+	989186	1079954	2748	10
Bf1OR40	F	83350	232	-	389861	397533	1200	6
Bf1OR41	F	64035	239	+	253346	254734	1389	1
Bf1OR42	P	97467	303	-	98885	966391	744	3
Bf1OR43	F	98789	328	+	882268	883650	1383	1
Bf1OR44	F	101068	374	+	799576	800433	858	1
Bf1OR45	F	101541	384	+	691805	693022	1218	1
Bf1OR46	F	130733	459	+	338120	364538	1146	15
Bf1OR47	P	186988	459	-	334774	335124	351	1
Bf1OR48	P	245724	459	-	329995	330369	375	1
Bf1OR49	P	245735	459	-	332252	332713	462	1
Bf1OR50	F	106555	538	+	36404	47912	2838	3
Bf1OR51	F	131506	548	-	374028	383187	9160	7

Gene name	F/P	Protein ID	Scaffold	Strand	First position	Last position	Length	Exon
Bf1OR52	F	108014	603	-	333688	335204	1517	1
Bf1OR53	F	109263	676	+	91666	93129	1464	1
Bf1OR54	F	109265	676	+	111031	112491	1461	1
Bf1OR55	P	109264	676	+	104098	107439	954	2
Bf1OR56	F	110793	803	-	25493	26950	1458	1
Bf1OR57	P	139778	868	+	77311	77862	552	1
Bf1OR58	F	111310	873	-	12075	13436	1362	1
Bf1OR59	F	111311	873	-	17361	18722	1362	1
Bf1OR60	F	257613	873	-	7860	8717	858	1
Bf1OR61	F	111465	895	+	80564	81700	1137	1
