# Supplementary material for: Amphioxus (Branchiostoma floridae) has orthologs of vertebrate odorant receptors
Source: BMC Evol Biol. 2009 Oct 5;9:242. doi: 10.1186/1471-2148-9-242 (PMC2764704; doi:10.1186/1471-2148-9-242)
Supplement: Additional file 2 — List of vertebrate odorant receptors and non-OR GPCRs used in the phylogenetic analyses. This file contains a list of sequences used in the phylogenetic analyses and the GenBank accession numbers where available. [file 1471-2148-9-242-S2.PDF]

**Sequence Label in Tree**	**Species**	**Accession Number**	**Annotation or Source**
B.belcheri OR	*Branchiostoma belcheri*	GenBank: AB182635	Olfactory receptor-related GPCR gene coding locus
C.auratus OR1	*Carassius auratus*	GenBank: AAC64071	Putative odorant receptor
C.auratus OR2	*Carassius auratus*	GenBank: AAC64072	Putative odorant receptor
C.auratus OR3	*Carassius auratus*	GenBank: AAC64074	Putative odorant receptor
Dr3OR10.21	*Danio rerio*		Olfactory receptor [12]
Dr3OR10.24	*Danio rerio*		Olfactory receptor [12]
Dr3OR10.8	*Danio rerio*		Olfactory receptor [12]
Dr3OR15.35	*Danio rerio*		Olfactory receptor [12]
Dr3OR15.41	*Danio rerio*		Olfactory receptor [12]
Dr3OR17.1	*Danio rerio*		Olfactory receptor [12]
Dr3OR21.16	*Danio rerio*		Olfactory receptor [12]
Dr3OR21.21	*Danio rerio*		Olfactory receptor [12]
Dr_OR108-1	*Danio rerio*	GenBank: DQ305987	Odorant receptor (OR108-1)
Dr_OR104-1	*Danio rerio*	GenBank: DQ305993	Odorant receptor (OR104-1)
Dr_OR106-1	*Danio rerio*	GenBank: DQ305996	Odorant receptor (OR106-1)
Dr_OR109-1	*Danio rerio*	GenBank: DQ306009	Odorant receptor (OR109-1)
Dr_OR115-1	*Danio rerio*	GenBank: DQ306039	Odorant receptor (OR115-1)
Dr_OR101-1	*Danio rerio*	GenBank: DQ306041	Odorant receptor (OR101-1)
Dr_OR137-1	*Danio rerio*	GenBank: DQ306044	Odorant receptor (OR137-1)
Dr_OR134-1	*Danio rerio*	GenBank: DQ306048	Odorant receptor (OR134-1)
Dr_OR128-2	*Danio rerio*	GenBank: DQ306059	Odorant receptor (OR128-2)
Dr_OR121-1	*Danio rerio*	GenBank: DQ306074	Odorant receptor (OR121-1)
Dr_OR113-1	*Danio rerio*	GenBank: DQ306079	Odorant receptor (OR113-1)
Dr_OR118-1	*Danio rerio*	GenBank: DQ306087	Odorant receptor (OR118-1)
Dr_OR111-1	*Danio rerio*	GenBank: DQ306093	Odorant receptor (OR111-1)
Dr_OR103-5	*Danio rerio*	GenBank: DQ306105	Odorant receptor (OR103-5)
Dr_OR102-2	*Danio rerio*	GenBank: DQ306110	Odorant receptor (OR102-2)
Dr_OR124-1	*Danio rerio*	GenBank: DQ306119	Odorant receptor (OR124-1)
Dr_OR125-1	*Danio rerio*	GenBank: DQ306121	Odorant receptor (OR125-1)
Dr_OR133-1	*Danio rerio*	GenBank: DQ306129	Odorant receptor (OR133-1)
Dr_OR112-1	*Danio rerio*	GenBank: DQ306144	Odorant receptor (OR112-1)
Dr_OR130-1	*Danio rerio*	GenBank: DQ306145	Odorant receptor (OR130-1)
D.rerio OR	*Danio rerio*	GenBank: ABC43280	Odorant receptor
Fr3OR142.7	*Fugu rubripes*		Olfactory receptor [12]
Fr3OR18.1	*Fugu rubripes*		Olfactory receptor [12]

Fr3OR2346.3	*Fugu rubripes*		Olfactory receptor [12]
Fr3OR6030.1	*Fugu rubripes*		Olfactory receptor [12]
Fr3OR633.1	*Fugu rubripes*		Olfactory receptor [12]
Fr3OR7011.1	*Fugu rubripes*		Olfactory receptor [12]
Fr3OR7149.1	*Fugu rubripes*		Olfactory receptor [12]
Gg2OR1.2	*Gallus gallus*		Olfactory receptor [12]
Gg2OR5.19	*Gallus gallus*		Olfactory receptor [12]
Gg2OR9.1	*Gallus gallus*		Olfactory receptor [12]
Gg2ORUn.61	*Gallus gallus*		Olfactory receptor [12]
H.sapiens OR2L5	*Homo sapiens*	GenBank: DAA04856	Olfactory receptor 2L5 (also called OR1-53)
L.fluviatilis OR	*Lampetra fluviatilis*	GenBank: CAA10136	Olfactory receptor
M.musculus Olfr683	*Mus musculus*	GenBank: AAP71150	Olfactory receptor Olfr683
O.latipes OR1	*Oryzias latipes*	GenBank: BAA84275	Olfactory receptor 1
O.latipe s OR2	*Oryzias latipes*	GenBank: BAA85093	E1 olfactory receptor
X.laevis OR1	*Xenopus laevis*	GenBank: AJ250750	Olfactory receptor class I (xb238 gene)
X.laevis OR2	*Xenopus laevis*	GenBank: AJ250753	Olfactory receptor class II (xb154 gene)
X.laevis OR3	*Xenopus laevis*	GenBank: AJ250754	Olfactory receptor class II (xb177 gene)
Xt1OR10411.1	*Xenopus tropicalis*		Olfactory receptor [12]
Xt1OR11576.2	*Xenopus tropicalis*		Olfactory receptor [12]
Xt1OR2286.1	*Xenopus tropicalis*		Olfactory receptor [12]
Xt1OR35679.1	*Xenopus tropicalis*		Olfactory receptor [12]
Xt1OR39249.1	*Xenopus tropicalis*		Olfactory receptor [12]
Xt1OR42821.1	*Xenopus tropicalis*		Olfactory receptor [12]
Xt1OR5329.1	*Xenopus tropicalis*		Olfactory receptor [12]
Xt1OR5508.1	*Xenopus tropicalis*		Olfactory receptor [12]
D.rerio melanocortin receptor 1	*Danio rerio*	GenBank: NP_851301	Melanocortin 1 receptor
H.sapiens melanocortin receptor	*Homo sapiens*	GenBank: AAC13541	Melanocortin receptor
M.musculus Taar7a	*Mus musculus*	GenBank: Q5QD12	Trace amine-associated receptor 7a
R.norvegicus Taar5	*Rattus norvegicus*	GenBank: Q5QD23	Trace amine-associated receptor 5
H.sapiens Taar2	*Homo sapiens*	GenBank: Q9P1P5	Trace amine-associated receptor 2
R.norvegicus Taar3	*Rattus norvegicus*	GenBank: Q5QD24	Trace amine-associated receptor 3
M.musculus Taar4	*Mus musculus*	GenBank: Q5QD15	Trace amine-associated receptor 4
D.rerio Taar66	*Danio rerio*	GenBank: NP_001076512	Trace amine-associated receptor 66
D.rerio Taar1b	*Danio rerio*	GenBank: AAI63112	Trace amine-associated receptor 1b
H.sapiens P2Y1 receptor	*Homo sapiens*	GenBank: NM_002563	Purinergic receptor P2Y (P2RY1)
D.rerio P2Y receptor	*Danio rerio*	GenBank: CAK04925	Protein similar to vertebrate purinergic receptor P2Y
H.sapiens P2Y11 receptor	*Homo sapiens*	GenBank: NP_002557	Purinergic receptor P2Y11

H.sapiens P2Y12 receptor	*Homo sapiens*	GenBank: NP_073625	Purinergic receptor P2Y12
H.sapiens hypocretin receptor 2	*Homo sapiens*	GenBank: CAH73407	hypocretin (orexin) receptor 2
D.rerio hypocretin receptor	*Danio rerio*	GenBank: ABL96925	hypocretin receptor
H.sapiens oxytocin receptor	*Homo sapiens*	GenBank: AAQ91333	Oxytocin receptor
G.gallus oxytocin receptor	*Gallus gallus*	GenBank: NP_001026740	Oxytocin receptor
H.sapiens opioid receptor delta 1	*Homo sapiens*	GenBank: NP_000902	Opioid receptor, delta 1
D.rerio opioid receptor kappa 1	*Danio rerio*	GenBank: NP_878306	Opioid receptor, kappa 1
H.sapiens somatostatin receptor	*Homo sapiens*	GenBank: AAA20828	Somatostatin receptor
H.sapiens somatostatin receptor 5	*Danio rerio*	GenBank: NP_998462	Somatostatin receptor 5
D.rerio alpha2B-adrenergic receptor	*Danio rerio*	GenBank: AAL07509	Alpha2B-adrenergic receptor
R.norvegicus alpha2B-adrenergic receptor	*Rattus norvegicus*	GenBank: AAK53388	Alpha2B-adrenergic receptor
D.rerio adenosine receptor A2a.2	*Danio rerio*	GenBank: AAI62249	Adenosine receptor A2a.2
B.taurus adenosine A1 receptor	*Bos taurus*	GenBank: CAA45135	Adenosine A1 receptor
M.musculus mas proto-oncogene	*Mus musculus*	GenBank: CAA47964	Mas proto-oncogene
H.sapiens mas-related GPR member X3	*Homo sapiens*	GenBank: NP_473372	Mas-related GPR, member X3
H.sapiens Fpr2	*Homo sapiens*	GenBank: AAH29125	Formyl peptide receptor 2
M.musculus Fpr-rs3	*Mus musculus*	GenBank: AAC34586	N-formylpeptide receptor-like 3
M.musculus Fpr-rs2	*Mus musculus*	GenBank: NP_032065	Formyl peptide receptor related sequence 2
M.musculus Fpr1	*Mus musculus*	GenBank: NP_038549	Formyl peptide receptor 1
D.rerio Fpr1-like	*Danio rerio*	GenBank: CAK05401	Protein similar to vertebrate formyl peptide receptor 1
B.floridae 225039	*Branchiostoma floridae*	Protein_ID: 225039	Rhodopsin-like GPCR [16]
B.floridae 227418	*Branchiostoma floridae*	Protein_ID: 227418	Rhodopsin-like GPCR [16]
B.floridae 107702	*Branchiostoma floridae*	Protein_ID: 107702	Rhodopsin-like GPCR [16]
B.floridae 202803	*Branchiostoma floridae*	Protein_ID: 202803	Rhodopsin-like GPCR [16]
B.floridae 211803	*Branchiostoma floridae*	Protein_ID: 211803	Rhodopsin-like GPCR [16]
B.floridae 148901	*Branchiostoma floridae*	Protein_ID: 148901	Rhodopsin-like GPCR [16]
B.floridae 92625	*Branchiostoma floridae*	Protein_ID: 92625	Rhodopsin-like GPCR [16]
