# Supplementary material for: Amphioxus (Branchiostoma floridae) has orthologs of vertebrate odorant receptors
Source: BMC Evol Biol. 2009 Oct 5;9:242. doi: 10.1186/1471-2148-9-242 (PMC2764704; doi:10.1186/1471-2148-9-242)
Supplement: Additional file 3 — Multiple sequence alignment used for the phylogenetic analyses. This file contains the multiple sequence alignment used to generate the Neighbor-Joining trees in Figure 1 and Additional file 4. The N and C termini have been removed as well as three blocks of sequence that could not be aligned (shown as gaps in the alignment). [file 1471-2148-9-242-S3.PDF]

```
                            10        20        30        40        50        60        70        80        90
                    ....|....|....|....|....|....|....|....|....|....|....|....|....|....|....|....|....|....|
Dr3OR10.21          FPHAKYYYLFLSLVYAVTVLGNSFIMCIIYLSRRLHTAKYIAVFHLALCDLCGSSAVIPKVVDTFLFD-CMANIFFVFHFMNVQSLTLLV
Dr3OR10.24          VPHAKYYYVFLSLAYVVTVLGNSFLMCIMYLSRRLHTAKYIAVFHLALSDLCGSSTVIPKVIDTLLFD-CLTHMFFIYHFMNLQSLTLLV
Dr3OR10.8           LQNQKLLGSLLLISYMFILIGNIINLCVISTDRHLHKPMYILICNLAVVDIMYSSTCSTTMISVLLAE-CITRTFFYHVADIAECMALTL
Dr3OR15.35          IPFSNYYYIFLFVIYFISVIGNSVVLLIIAVDRSLHSPKYIGVFNLALADIGETNALIPNMMKTFLFD-CLANMFFVFLFSSVQSFTLVA
Dr3OR15.41          IPYSSYYYVFLFITYFITIIGNSVVLLIIAFERSLHSPKYIVVFNLALADIGETNALIPNMMKTFLFD-CLVNMFFVFLFSTMQSFTLVV
Dr3OR17.1           GDIKYLCFVVLILLFAAIIVANLFIIGIICKEKSLHEPMYVFLCSLCANQLCGSFALFPFVLGNMLRS-CHAQIFALYTYGQVEFCNLAM
Dr3OR21.16          KSYRHVYFTCFLLLYLLILSINIRLVMVIIMEKALHGPMYIFLCHLCVNGVYGATGFYPKFLSDLILD-CALQTYVIYSSLLSDITILTV
Dr3OR21.21          QFAPVRTFLIVTPCVLFLYVNAVMIFTLRKKTVFQQTSRYILFGQMLWVDTLNLFMSVVMYVCAASGI-CLLLLVSATALYQISTLNLSL
Fr3OR142.7          PDYYGHVSAALFLVLSAIVVGNVFILVFVKCERSLHKPTYLIFCHLASTDLLFGLVTLPKIISKYWFG-CFVQMYFVHFLGATHSFVLMV
Fr3OR18.1           SYTCVRFYVSTASFTLLLLFNFVINSTIVRVEHLRSHARFVLVFHLLLSALVYLGMSSIFHYQVYLRA-CQAMVAVLITSGSNILLTLTV
Fr3OR2346.3         VKYRNTLFALTFLCYFLIIVVNICLILTIIIEKKLHEPMYIFLGSLCFNGLYGATGFYPKFLSDLLSD-CFVQIYVIYSNAKIDYSILVV
Fr3OR6030.1         LERMMFSSLTTVPCCVFLYINGIMLFSLRSKTVFCETSRYILLFNLLFADSIQMVLGQALYILAVCLI-CGILTALGVLTSNISPLTLVV
Fr3OR633.1          WHLEYFFIPSTVVTLATLVANPVLLICIFLTRALRQETRYLLVANTLAADMLFLILNLVTLICNTVTA-CELVTALTVTSYCCAILTVTL
Fr3OR7011.1         ARIFEQIIITQLIVSIFFMINLLLIATVFSNQVFLSTMRYILFLITLVSDSVFLILSDLLLLLYSIYF-CIIFYLIVCQCMFVTPVTLTA
Fr3OR7149.1         GELKYLYFGIMLFWFMSIWMVNSVLILIIYKDKRLHEPMYIFVCHLFVNEISGSTSLYPLMLSQMLSD-CFLQMCYIYTSASLEFCSLAA
Gg2OR1.2            EESHIWISVPFCLMYITALISNSVLLFVIATERSLHEPMYLFLAMLAVSDLMLSTTTVPKMLAIFWFS-CITQMFFTHFSFIVESSVLLA
Gg2OR5.19           TELQVPLFFIFSVIYTIAVLGNLGMIVLIWTHLQLHTPMYFFLGNLSVIDLCSATVFAPRMLGNFFAQ-CLSQTFFFVVFITTEVFLLTA
Gg2OR9.1            TNVKMFLIPPVVCLVAGVLIIPSILFVIFSRLSIRQETRYMLLGNALLCDLLYLLLYTLSAALSAAHV-CILQLFSLAVAYCGGLFTAAA
Gg2ORUn.61          EKEQFWIAFPFCIMYAIAVLGNIILLIIIKTESSLHEPMYLFLAMLAFTDLVLSTSILPKMLIIFWRG-CLVQLFFVHSFTTIESGVLVA
C.auratus OR1       LENKNYLILALAIIYIITLLGNLVLLAVVLMNSSLQSPKYLAVCNLAIIDISINSVIIPQMVPVFAFN-CFSQMFFMHFFGDMESFSLAL
C.auratus OR2       PGYYGAVAALLLFAYVCILVGNGIFIALFTLEKRLHKPMYNIILNLVVSDLLFSTTTLPKIISRYWFQ-CFIQMYFVHYFGSVSSVILAI
C.auratus OR3       MGKRFMFFSLTALYYPFIVLCNVIIIYTVISHKKLHEPMYVFICNLCMNGLFGTAGFYPKFMYDLLSQ-CLVQIFVIYSSVLCDFSTLTV
L.fluviatilis_OR    PIVKFAVFFFTLAMFTAALMGNCLLSLVILRDHQLHKAMYVLMAALSLTDTISALSILPRFMQNVASF-CIAQMFFLHLAIRMQSFTLTI
B.belcheri OR       STSHGLQTTYLIISLVLSVGCGLLLIFLVWKKENLRKPSHYLRCNLAVDDIIFTSCLIPIRIYALFEP-LGKSTGITPVCAMSMFGTYLM
O.latipes OR1       EKYRCLYFLIVFTVFILILFCNCTIIFIIVVQKSLHEPMYIFIAALLTNSVMFSMAVYPKLLVDILSK-CLLQYVVYYCLGASEFLLLTA
O.latipes OR2       IPNVDYYYIFLLFVYIVSVAGNTTVMALIYLDHNLKTAKYVAVFNLAFVDLLGSTALVPKVIDIFLFD-CMTFLFFCFTCLSMQALNLVA
Dr_OR108-1          VPYSNVYVMFLTVLYVITVICNVFLMSIIVYDQRLHVPKFMAVGNLAVVDLILSTSLVPGMIKIYIVL-CLVQMYTYYTFLELETLSLCI
Dr_OR104-1          PKYYSLTAALLFCIYIAVITGNSLIVVLFVIERSLHKPMYIIMLSLSVSDICFCTVALPKVISRYWFN-CLFQRQLIHYFGTLNSLIMML
Dr_OR106-1          LQNQKLLGSLILITYILVLLGNGINLCVISTNRHLHKPMYILICNLAVVDVMYSSTCSTTMISVLLAD-CISRMFFYHIGDFTECMALTL
Dr_OR109-1          MPQAKYYYMFLSLVYAVTVLGNSFIMCIIYLARRLHTAKYIVVFHLALSDLCGSSALIPKIIEMFLFD-CLTNMFFVYHFMNLQSLTLLA
D.rerio OR          LVTSTKTAVAVLTSLFFFFVNCVMFFALKSKHIFFETPRYILFGHMLMNDSVLLLVTTVMYVVALCFL-CTLLVFISHCTFRNAPLTLAL
Dr_OR115-1          PQSSYPVFILLLLVYVFIMVSNIGLIVLISTEKNLHHPMHFLFCNLPLNDIIGTNVILPRLMQDMLRD-CVVQAYFVHLFAIACHYVLII
Dr_OR101-1          GLKDKIVLPIFFTVYVSVLSGNSMIIYLVRTDPKLDTPMYFFLHNLSFSDIVYTSVTIPKMLSVFLTG-CFMQIQFVLWMGLTGRGLLTA
Dr_OR137-1          SDVFAQNFTVVCLAVIIISINGMFVFIFFRRSIFHSDPRYILYIHLVINDMLMVFTSVILFVMAFIWN-CVTLLIIASTAYKNTPLTLAG
Dr_OR134-1          AMAFMKNFIVVLVWFLLSYINGSVVATFFRHQIFYEDPRYILFIHMVINDAVQLTVTIALFLVSYILY-CCFFILVAVFTTRSTPVNLAG
Dr_OR128-2          GYIRYALFILGFVLYFAIILFNVLIMLAVFRERTLHQPMYILISCLSMNSLFGTAGFFPRVLSDLLSE-CFFQSFVIFTYAANEFTILML
Dr_OR121-1          FKMKYFLFAVLTVLYSTIIFANSFLIGVICLAKILHKPMYMFLCSLFVNELYGSTALFPSLQVNLLLN-CYLQIFCLYTYAMIEFCNLAV
Dr_OR113-1          GEWRPFLFIPFFLMFMLAIIANSILIYLIKTQRSLHSPMYVLIGVMAVVDLIMPLSFVPNMLLSFLFN-CLMQMFGIHFVGSFQVTLLFW
Dr_OR118-1          EANIYPAFIFGLTTYLFILLCNLTIIITICLNRNLHKPMYILLLNLPINDVLGATCLFPQLLYSIWSQ-CLLQGFFVHLNGGASYLILTA
Dr_OR111-1          IGYSSYYYIFLFITYFITVVGNTAVILIIVLDRRLHSAKYIGMFNLALADFGEANALIPNMMKTFLFD-CMANMFFVFLFSTMQSFTLVV
Dr_OR103-5          PHYYGLVSALLFFVYVFTLIGNAVFFTLFVITKSLHKPVYYFIINLVVSDVLFSTATLTKIISRYWFQ-CFVQMFFVHLFGSVCSVVLAV
Dr_OR102-2          PEYHGSVGALLFILYLVLATGNIFIIVFVMIEKSIQKPTYIIFCNLAMADLALGTTTYPRVIAKYWMS-CFTQMFFIHFLGATNSFLMAL
Dr_OR124-1          WTSRILFFSFALPGYLLTIILNATLIMIIAFEKALHEPMYIFLCNLCINDLCGITGFYPRALTYLLTE-CIVQGLVIVIYATGEFTSLSV
Dr_OR125-1          KLYRHIYFICFLVLYLLILSINICLVMVIIIEKVLHEPMYIFLCHLCINGVYGATGFYPKILSDLILD-CALQTYVIYSSLLSEITILTV
```

```
Dr_OR133-1      VDRDLKTILSVSSCVIFLYVNGVMIFTLRKKPVFQETSRYILFGHMLWVDTLNLVMSVVLFACAVFRP-CLILLVAATALYQVSTLNLAL
Dr_OR112-1      YKYRRFLFLPFFLIYLLLLVGNSLLLFVIKSTESLHSPMYIFVSALAVVDIIVPTAIIPAMLLGFLFD-CLTQMFVTHFFSSVESTILLA
Dr_OR130-1      VRRNISLALTQVIVWPFIYLIFLMLLIFSKKETFTTEMRYILFGHSLLVDLTFLLLTDFVVLLSYNLV-CIPVCMFIETVAVSAPLTVGA
X.laevis OR1    LNFFLPVSITMFVAYIVSLIANSTVIILIILREQLHHPMYIIIANLALSDLLFDTITLPKIIAKYWFG-CFFQLFCVHSLGSLDSFIIML
X.laevis OR2    PDLQTPLFLIFLLSFLIIIIGNTSVFATIILSVPLHKPMYVFLGSLSVLDISSASTTLPNLLAMLHTQ-CILQFYFFMAFAITEFILLSA
X.laevis_OR3    HNFKIPLFSLFLLIYLLIIWENVLIIVLVLYNRNLQSPMYFFLQQLSLTDLLESTNIVPTLLQTVIND-CITQLYFWGVSETFECFLLAV
Bf1OR44         PASQGLQTAYLVISLIVIVAANFLIILLVCRKEYLRQPRYFLRCHLAVNDTLFALVIIPRYLDLILQS-CSDVRIFTSAVALSKYGIYLL
Bf1OR45         SASLGLQTAYLIFSLVVAVGCGLLVVYLVYKKDYLQKPSNYLRCSLSAYDIIFMCCMIPMDIYVLYQA-CWVREQLFRWFTTSMSGTYVL
Bf1OR50         LVAKIFQVCWLVPSLVLILGLNVIFIITVLRSPSLWKPRFLLSVNLAILDILLSLVFIPSSVHNIVSE-CLTQCSVFFGIAICTASTLLV
Bf1OR52         SASLGLQTTYLILSLVVAVGCGLLVIYLVFKKDYLQKPCNYLRCSLASYDIVFMCCMIPTDIYMIFQT-CWMRGQMFGWFGASMGGTYLL
Bf1OR53         STFRDLQTAYLIISLVLSVGCGLLLIFLVWRKDYLQRASNYLRCNLAVDDIIFTSCLIPFRIYALYRH-CSAVVVVSSPCLLSMSGTYLM
Bf1OR54         STFRDLQTAYLIISLVLSVGCGLLLIFLVWRKDYLQKPSHYLRCNLAVDDIIFTSCLIPFRIYALFRL-CSAVVLVVNPCLLSMSGTYLM
Bf1OR56         PLSRGLQTLYLVTSLVVAVGCSSLVIFLVWKTESLQKPRHFLRCNLALDDIIFTGSMIPLEIYGLFVD-CGIQNTPIPTFTASMFGAYLL
Bf1OR58         TLSLGLQTAYLIISLVLAVGCSLLLIYLVYKKDFLQRPRHYLRCNLSIDDIIFTVFLIPTELCLLFSQ-CWVQILVGQLSSSSMFGTYFL
Bf1OR59         TLSLGLQTAYLIISLVLAVGCSLLLIYLVYKKDFLQRPRHYLRCNLSIDDIIFTVFMIPTELCLLFSQ-CWVQMLAGRLSSSSMFGTYFL
Bf1OR61         STSLGLQTAYLIFSLVVAVGCGLLVLYLVYKKEYLQKPSNYLRCSLAAYDIIFMCCVVPTDIYLSFQT-CRLRRQLFGFFASSMSGTYVL
Bf1OR12         LLGKLLQLFWLVPSLVLILSLNCLFLVAILRTKKLWRPRFVLPASLCVLDIVQGSLFIPSSVVNIVMG-CWIQASYLYVGAVCTLSTLTL
Bf1OR37         FYVGFYDNEKVKTLGDNSEELTVLVGPGVWKKVYLRKPRHFLRSNLAVDDMVFVACLIPLEICLLFSQ-CWLQLMVAYPTTASMFGTYLM
Bf1OR46         LMGKLLQLFWLVPSLVLILSLNCLFLVAILRTKKLWRPRFVLPASLCVLDIVQGSLFIPSSVVNIVIG-CWIQASYLYVGAVCTLSTLTL
Bf1OR51         FYVGFYDNEKVKTLGDNSEELTVLVGPGVWKKVYLRKPRHFLRSNLAVDDMVFVACLIPLEICLLFSQ-CWLQLMVAYPTTASMFGTYLM
Bf1OR28         TLSLGLQTAYLIISLVLAVGCSLLLIYLVYKKDFLQRPRHYLRCNLSIDDIIFTVFLIPTELYLLFSQ-CWVQILVGRLSSSSMFGTYFL
Bf1OR60         TLSLGLQTAYLIISVVLAVGCSLLLIYLVCKKDFLQRPRHYLRCNLSVDDIIFTVFLIPTELCLLFSQ-CWVQILVGRLSSSSMFGTYFL
Bf1OR41         STFRDLQTAYLIISLVLSVGCGLLVIFLVWKKEHLRKPSHFLRCNLAVDDIIFTSCMIPVRIYALFRE-CSGRMLVGAPCLVSTYGTYLM
Bf1OR11         PVARGFQIAYLVTTVALSVVCGILVIVLVWKKEYLQKPCHYLRCNLAVDDVLFTGLCVPNVIHNISQG-CRVEGAFTLGLLLSKTCTYLL
Bf1OR1          SLARGLQVSYLAISVALSVFCGLLVIVLVWAKEYLQKPCHYLRCNLAVDDVLLTGVYVPTVIHNIRQG-CRAEAVLVNNLLLSKTFAYLL
Bf1OR2          PVARGFQIAYLVTTVALSVVCGLLVIVLVWKKEYLQKPCHYLRCNLAVDDILFTGLCVPNVIHNISHG-CRVEGAFTLGLLLSKTCTYLL
Bf1OR13         LLGKLLQLFWLVPSLVQILSLNCLFLVAILRTKKLWRPRFVLPASLCVLDIVQGSLFIPSSVVNIVMG-CWTQASYFYVGACCTLSTLTL
Bf1OR14         LLGKLLQLFWLVPSLVLILSLNCLFLVAILRTKKLWRPRFVLPASLCVLDIVQGSLFIPSSVVNIVIG-CWTQAGYFYVGACCTLSTLTL
Bf1OR5          STFRDLQTAYLITSLVLSVGCGLLLIFLVWRKEHLQRPSNYLRCNLAVDDIIFTSCLIPIRIYALSRQ-CSAWLVVGTPCLTSISCTYLM
Bf1OR38         RLSRGLQTAYLIISLAVSVGCGLLLIYLVCTKGYLQKPRHFLRCNLAIDDIIFTSCIITTEICFLFSQ-CWVQVLVVHPSTISMFGTYLL
Bf1OR8          STSQTLQVTYLIISLVLSVGCGLLLIFVVWKKRYLHKSSHYLRCNLAVDDIIFTSCLIPIRIHALLRQ-CTARSLIAPPCQTSMAGTYLM
Bf1OR9          STSRTLQVTYLIISLVLSVGCGLLLIFVVWKKGYLHKPSNYLRCNLAVDDIIFTSCLIPIRVYALLQQ-CKARLLVGPPCVLSMCGTFVM
Bf1OR10         STSQTLQVTYFIISLVLSVGFGLLLIFVVWKKRYLHKPSHYLRCNLAVDDIIFTSCLIPIRIYALLQQ-CTARSLVGLPCLASMVGTYLM
Bf1OR4          ATSQTLQVTYLIISLVLSVGCGLLLIFVVWKKRYLHKPSHYLRCNLAVDDIIFTSCLIPIRIHALLRQ-CMARLLVGPPCLTSMAGTYLM
Bf1OR19         STFRDFQTAYLIISLVLSVGCGLLLIFLVWRKDYLQKPSHYLRCNLAVDDIICTSCLIPFRIYALFRL-CSAVAVIVNPCLLSMSGTYLM
Bf1OR15         STSRDLQTTYLVISLVLSVGCSLLLIFLVWKKEYLQRPGNYLRCNLAVDDIIFTSCLIPFRIYALFRQ-CSARMLVAPPCLTSMGGTYLM
Bf1OR26         SASLGLQTTYLILSLVVAVGCGLLVIYLVFKKEYLQKPCNYLRCSLASYDIVFMCCMIPTDIYMIFQK-CWMRGQMFGWFGASMGGTYLL
Bf1OR30         STFRDLQTAYLIISLVLSVGCGLLLIFLVWKKENLQKPSHFLRCNLAVDDIIFTSCLIPIRIVALFRQ-CSGRVLVAPASLPSMSGTYLL
Bf1OR18         STFRDLQTAYLIISLVLSVGCGLLLIFLVWKKHHLQKPSHFLRCNLAVDDIIFPSCLIPIRIVALFRQ-CSGMALVAPASLLSMIGTYLM
Bf1OR27         TLSLGLQTAYLIISLVLAVGCSLLLIYLVCKKDFLQRPRHYLRCNLSIDDIIFTVFMIPTELCLLFSQ-CWVQMLTGRLSSSSMFGTYFL
Bf1OR24         STSRDLQTAYLITSLVLSVGCGLLLIFLVWKKESLQKPSYYLRCNLAVDDVIFTSVMIPIRIYALFQQ-CIGRTLVVPTCVLSMFGTYLL
Bf1OR23         STFRDLQTTYLVISLVLSVGCGLLLLFLVWKKQHLQRPSNYLRCNLAVDDIIFTSCLIPIRIYALFRQ-CSAKAVVGPVCLLSMFGTYLM
Bf1OR29         STFRDLQTTLLIISLVLSVGCGLLVIFLVWEKHYLQKPSHFLRCNLAVDDIIFTSCLIPIRIYALFRQ-CSGMALVAPTCIVSMSGTYLL
Bf1OR22         LTAKILQVCWLVPSLVLILGFNVVFIIAVARSPSLWKQRFLLPVNLAVLDILLALVCIPSSINNIVSE-CLTQCGVYYGTAVCTLMTLTL
Bf1OR40         LTAKILQVCWLVPSLVLILGFNVVFIIAVVRSPSLWKQRFLLPVNLAVLDILLALVCIPSSVNNIVSE-CLTQCGVYYGTAVCTLMTLTL
Bf1OR25         -------MCWLVPSLVLILGLNAIFILAVVRSPSLWKPRFLLPVNLGVLDILLALVFIPSSLHNIVSE-CLTQCGVYFGTAECTMATLLL
```

```
Bf1OR31              PTSKGFQIAYDAVCLVVAVVCNTLVIFLVCKTESLHKPRHYLRCHLAAVEIVFATILIPFNIETIVNG-CELLYIIGNLSGIAIFGTYFL
Bf1OR36              LTAKILQVCWLVPSLVLILGLNAIFIVAVVRSPSLWKPRFLLPVNLGVLDILLALVFIPSSLHNIVSE-CLTQCGVYFGTAECTMATLLV
Bf1OR3               STFRDLQTAYLIISLVLSVGCGLLLIFLVWKKESLQNPSNYLRCNLAVDDIIFTSCLIPIRIVALFRQ-CLGRTLVAPSCLLSMFGTYLM
Bf1OR32              STFRDLQTAYLIISLVLSVGCGLLLIFLVWKKESLQNPSNYLRCNLAVDDTIFTSCLIPVRIFALFRQ-CLARTLVAPSCLLSMFGTYLM
Bf1OR34              RLSRGLQTAYLIISLAVSVGCGLLLIYLVCTKGYLQKPRHFLRCNLAIDDIIFTSCIITTEICFLFSQ-CWIQVLVVHPSTISMFGTYLL
Bf1OR17              -----------------MVRIRLDKTTRTGTRTQIERAWAALAQPSAVDDIIFTGCLIPFRIYALFQQ-CSARMLVAPPCLTSMAGTYLM
Bf1OR39              STFRDLQTTYLIISLVLSVGCGLLLLFLVWKKDYLQKPSHFLRCNLAVDDIIFTSCLIPVRIYAIFQQ-SAAKLLVAPPCLLSMFGTYLM
Bf1OR6               PLEQALQVTYVTLSYLATLGGNIGVLVVILSYKRLRKPRYYVHCSLACCDIFITTVCIPTVIVNLLYR-CQVHSAVYMTMSACTLFNLAI
Bf1OR21              PASQGLQTAYLVISLIVIVAANLLIILLVCRKEYLRQPRYFLRCHLAVNDILHALVIIPRYLDLIMQS-CSDVRIFTSAVALSKYGIYLL
Bf1OR43              STFRDLQTAYLIISLVVSVGCGLLVIFLVWKKEHLRKPSHFLRCNLAVDDIIFTSCMIPVRIYALFRE-CSGRKLVGAPCLVSTYGTYLM
H.sapiens OR2L5      SKIGLFLFILFVLIFLMALIGNLSMILLIFLDTHLHTPMYFLLSQLSLIDLNYISTIVPKMASDFLYG-CGIQSFFFMTFAGAEALLLTS
M.musculus Olfr683   QTWQHWLSLPLSLLFLLAMGANATLLITIRMEASLHEPMYYLLSLLSLLDIVLCLTVIPKVLAIFWFD-CFLQMFVMNSFLTMESCTFMV
Xt1OR10411.1         HSLRIQFFLAILILYIFTISGNAMVAALVLSSHSLQQPMFLFLGHLSLCDITLTTTIVPVLLHGLLRG-CLTQFQIFFAGVASECFLLGV
Xt1OR11576.2         ERFNYLYCALSLLAYLFILLCSTIIILVVLQEESLHEPMYTLIANLVLNGIFGSSTFFPKLTVDLLLS-CLIQSFCVLFFGYCEISLFAI
Xt1OR2286.1          SQSRHWLFIPFFFIYLEILMGNFMIMYRIWVEKSLHFPMYSLICLLFAVNISCTTAIVPNMLMGLAFG-CLFQMFFIYTAVILESTVLMI
Xt1OR35679.1         PNFFLPVSLTLFLAYIVSLIANSTVIILIILREQLHQPMYIIIANLALSDLLYDTITLPKIIAKYWFG-CFFQIYCVHHLGCLDSLIIML
Xt1OR39249.1         AGIKYAYSTLIFLCFAVIAASNCAVVGTIVAHRSLHEPMYILIAALSVNGLYGSAAFFPNLFINLLSK-CIIQMFGLHTYVGCEMAILAV
Xt1OR42821.1         EPFHTWISIPFCSIFLIAVIGNLVVLQIIISEVSLHQPMYIFVTVLSIIDLVLANSTMPKLLSIFWSS-CLFQMFLLHAFSAIESGIFVA
Xt1OR5329.1          IVLLLLIIFILLCFCIFLYIMAAILKVFFTTPHVRENVRYVLFIHMLITDSLYLFLSIFVFISAVYMV-CFAIVILVTTAFVVTPYNLAV
Xt1OR5508.1          LTETVKTCIYLVDFLICSVFAIVIIRTISSHSVLKKEVRFFLLCHHLTYLTLFFAFGTIYNSVRASKP-CWTLFAVLIVCGRVLLMTLVL
M.musculus Taar7a    PGPRLILYAVFGFGAALAVCGNLLVMTSILHFRQLHSPANFLVASLACADFLVGLTVMPFSTVRSVEG-CKFHSCFEGSFCYSSIFHLCF
R.norvegicus Taar5   LAIRVLIYLACAVGMLITVLGNLFVVFAVSYFKVLHTPTNFLLLSLALADMLLGLLVLPLSTVRSVES-CRLHTYLDTLFCLTSIFHLCF
H.sapiens Taar2      LGVRVAMYSFMAGSIFITIFGNLAMIISISYFKQLHTPTNFLILSMAITDFLLGFTIMPYSMIRSVEN-CKIYYSFDLMLSITSIFHLCS
R.norvegicus Taar3   FRVRLIMYLLMTGAMVITIFGNLVIIISISHFKQLHSPTNFLILSMATTDFLLGFVIMPYSMVRSVES-CKFHASFDMMLSLTSIFHLCS
M.musculus Taar4     ALVVCAMYLIMIGAIVMTMLGNMAVIISIAHFKQLHSPTNFLILSMATTDFLLSCVVMPFSMIRSIES-CKVHSCCDIMLCTTSIFHLCF
D.rerio Taar66       VSTQTVVYLVLASAMTVTILGNSVVIISIAHFKQLQTPTNILVMSLALADLLLGLVVMPFSMIRSVDG-CLLHSSFDMFLTSVSIFHLIF
D.rerio Taar1b       HAIHVPMLIAIMLIISMTFIGNLLVIISIGHFRQLHTPTNQLILSLALCDFLIGLFVMPLSAVRSMQG-CKLHTCIDITLSTSSIFHLVS
H.sapiens P2Y1 recep GFQFYYLPAVYILVFIIGFLGNSVAIWMFVFHMKPWSGISVYMFNLALADFLYVLTLPALIFYYFNKT-CKLQRFIFHVNLYGSILFLTC
H.sapiens P2Y11 rece FQGDFLWPILVVEFLVAVASNGLALYRFSIRKQRPWHPAVVFSVQLAVSDLLCALTLPPLAAYLYPPK-CRLERFLFTCNLLGSVIFITC
H.sapiens P2Y12 rece YKITQVLFPLLYTVLFFVGLITNGLAMRIFFQIRSKSNFIIFLKNTVISDLLMILTFPFKILSDAKLG-CQVTSVIFYFTMYISISFLGL
D.rerio P2Y receptor EFKYILLPVSYSLVCFFGLILNSVALWMFITKMRPWKPSTVYMFHLALSDTLYVLSLPMLIYYYANRS-CKIVRFLFYANLYCSILFLTC
H.sapiens hypocretin KEYEWVLIAGYIIVFVVALIGNVLVCVAVWKNHHMRTVTNYFIVNLSLADVLVTITCLPATLVVDITE-CKVIPYLQTVSVSVSVLTLSC
D.rerio hypocretin r KQYEWVLIAGYILVFLVSLVGNTLVCFAVWKNHHMRTVTNYFIVNLSFADILVTITCLPASLVVDITE-CKILPYLQTISVSVSVLTLSC
H.sapiens oxytocin r EALARVEVAVLCLILLLALSGNACVLLALRTTRQKHSRLFFFMKHLSIADLVVAVFQVLPQLLWDITF-CRLVKYLQVVGMFASTYLLLL
G.gallus oxytocin re EDMAKVEVTVLCLILFLALSGNLCVLLAIHTTRQKHSRMYFFMKHLSIADLVVAVFQVLPQLIWDITF-CRLIKYLQVVGMFASTYMLLL
H.sapiens opioid rec LALAIAITALYSAVCAVGLLGNVLVMFGIVRYTKMKTATNIYIFNLALADALATSTLPFQSAKYLMET-CKAVLSIDYYNMFTSIFTLTM
D.rerio opioid recep SPLIPIITAVYSVVFVVGLVGNCLVMYVIIRHTKMKTATNIYIFNLRVADALVTTTMPFQSTDYLLNS-CKVFISIDYYNMFTSIFTLTM
H.sapiens somatostat GARAVLVPVLYLLVCAAGLGGNTLVIYVVLRFAKMKTVTNIYILNLAVADVLYMLGLPFLATQNAASF-CRLVMTLDGVNQFTSVFCLTV
D.rerio somatostatin EESTKALAVIYLVVFIVGLTGNSLAIFVVLRYTKMKTVTNMYILNLAVADELYILGLPFLTTHNVLSY-CRILMWADSISQFTSTFCLTV
D.rerio alpha2B-adre PEATAAFATAITLMMLITIVGNILVIIAVLTSRSLRGPQNLFLVSLAAADILVATLIIPFSLANELMG-CEIYLALDVLFCTSSIVHLCA
R.norvegicus alpha2B VQATAAIASAITFLILFTIFGNALVILAVLTSRSLRAPQNLFLVSLAAADILVATLIIPFSLANELLG-CEVYLALDVLFCTSSIVHLCA
D.rerio adenosine re -MSSLVYIVLELVIAVLAVAGNVLVCWAVCLNSNLQSITNFFVVSLAVADIAVGVLAIPFAVTISIGF-CLFIACFVLVLTQSSVFSLLA
B.taurus adenosine A SAFQAAYIGIEVLIALVSVPGNVLVIWAVKVNQALRDATFCFIVSLAVADVAVGALVIPLAILINIGP-CLKVACPVLILTQSSILALLA
H.sapiens melanocort SLGIVSLLENILVILAVVRNGNLHSPMYFFLCSLAVADMLVSVSNALETIMIAIVHSDYLTFEDQFIQ-DNIFDSMICISLVASICNLLA
D.rerio melanocortin MLGLISLVENILVVVAIIKNRNLHSPMYYFICCLAVADMLVSVSNVVETLFMLLTEHGLLLVTAKMLQ-DNVIDIMICSSVVSSLSFLCT
B.floridae 225039    IRSPAREYVVLCIGVWTVVANSLPLAAIVKHEQLHTPVYILMAINLAASDVLTGATVVILFLLIHTYT-LHFIFTSAFLTGLSTAFGLLA
```

```
B.floridae 227418       -TWLVLTGLALSLLILVAILGNAMVLMAFIKDRRLCTPANYLIVSMAVADLLVSVSVMPLNATYELME-CDLWISLDVTSCTASILSLCV
B.floridae 107702       VRLGTGTDIVFSLIGLIEIIANAVVILGIIGTKELRKPIYFFLANLAMADVFAGIGLLYRTVGHVGHN-SVTYLNFLIFSQMTSASALSL
B.floridae 202803       AAVTGLIIMSYALVFLLCVVGNVIVCGVVIKTPRLRTVTNYFILNLAVSDLLVAIFCMPFTLVEHILP-CRVTPMIQGISVAASAYTMTA
B.floridae 211803       EPVVALFVLFYVFNFCLCIVGNTMVCYVILKIPRMQTVTNFFLLNLAVSDLLVAVFCMPFTLVDNIIR-CKLTPAVQVVSVAASVFTLVA
B.floridae 148901       ------MPIVFYVIFVVGILGNSVVMYVMIRHTKLRTPSDIFIFNLALADELFLIGIPFFAQQFISDE-CKIVYTLDSNNQFASVYILTT
B.floridae 92625        PESIVIPVIFAFIFCIGVVGNALVIVVLLRTGRELENTTNIFILNLSIADLLFIVFCVPFQAAVFTLP-CKLVHFFQKATMLASAFNLMS
M.musculus mas proto    HPPIPIVHWVIMSISPLGFVENGILLWFLCFRMRR-NPFTVYITHLSMADISLLFCIFILSIDYALDY-IVTLSVTFLFGYNTGLYLLTA
H.sapiens mas-relate    CYKQTLSFTGLTCIVSLVALTGNAVVLWLLGCRMRRNAVSIYILNLVAADFLFLSGHIIRSPLRLINI-SKILSPVMTFPYFIGLSMLSA
H.sapiens Fpr2          YTVLRILPLVVLGVTFVLGVLGNGLVIWVAGFRMTRTVTTICYLNLALADFSFTATLPFLIVSMAMGE-CKLIHIVVDINLFGSVFLIGF
M.musculus Fpr-rs3      SRVLWILSVIVLSITFVLGVLGNGLVIWVAGFRMAHTVTTICYLNLALGDFSFMVTLPLHIISMVMKG-CKFVLSIVHINLFVSVFLITL
M.musculus Fpr-rs2      SRVLWILSMVVVSITFFLGVLGNGLVIWVAGFRMPHTVTTIWYLNLALADFSFTATLPFLLVEMAMKE-CKLVHIVVDVNLFGSVFLIAL
M.musculus Fpr1         YIVLDVFSYLIFAVTFVLGVLGNGLVIWVAGFRMKHTVTTISYLNLAIADFCFTSTLPFYIASMVMGG-CKFIYTVIDINLFGSVFLIAL
D.rerio Fpr-like        EAIMNKVSTVFLTIIILFGTTGNSVVIWVAGFRMKPNVTNVFLVNLAAADLIFCLTQISWLIKDIFFD-CKFNGFVKYANMFCSVFLLAV

                                100       110       120       130       140       150       160       170       180
                        ....|....|....|....|....|....|....|....|....|....|....|....|....|....|....|....|....|....|
Dr3OR10.21              MAYDRLVAICFPLRYHAIVTKTSMLLILGVIWTFSLIFFSVFVDSI-HAVDRTKAIKTCTTHLMLVAIFYIPILSNNIAALT-DIQIINN
Dr3OR10.24              MAYDRLVAICFPLRYLAIVTKPSMFLIVGVMWIFSVIYFSVLVGLV-HGVDRIKAMKTCTSHLMLVAIGYLPLISNQIAALT-NTRIFNN
Dr3OR10.8               MAIDRVVAIRLPLRYHSIVTNLRTFCFIFVTWLMGFAAVGFFTSVL-SNSSRKQMINTCLSHLIVLLSYYAPKVVTILLTRI-TDRNAIL
Dr3OR15.35              LAYDRFIAICLPLRYHAIVTNTSMILIFSTIWVFNSSVVASMVSMI-TWEGRLKALKTCVSHLLLVGIFFLPIYCTYIAQVL-NARIIGT
Dr3OR15.41              LAYDRFVAICLPLRYHAIVNNSSITLILSAKWAFNSSMVALMVSLI-TWESRLKALKTCVSHLLLVGIYFLPMSCTYIAAWL-NARVITT
Dr3OR17.1               MSYDRYVAICHPLEYHRIMSPARTMTLIALGWSLQIIIFSLSFSVN-KGQTAQKSLYTCILHLISLLNFAVGCSFELIQSRF-VVRVILS
Dr3OR21.16              MSYDRYVAICKPLNYHSKLTKLTCAILILLSWIVPNFSVIPAIMLA-SLENRKKFWQTCLPHLFSLMNLIFTLLFDVTYSRY-SLRSFLA
Dr3OR21.21              MSLERYVAICFPLRHVELTSFERTRLAIGVVCIISMIQPLSETIIF---MSAKKANKTVLLHLLQLGFGVTSILFGVVQEVI-NVMYVGF
Fr3OR142.7              MALDRFIAVCMPLRYTVFITSATVSVLCGISWFMPMSWMVGIVLHA-DSSSRIRTLSTCTPQLLITCLYYMPRCFVYLANNL-PIRIVVV
Fr3OR18.1               MALDRCCAVCYPMRYTSYSTTWWWWPWLLGGLTWIMALAIALSLFL-INRRNLAGFKTITLHGTQLGVFILPNFVNFVLTML-ELSAVII
Fr3OR2346.3             MAYDRYVAICRPLEYHCVMSKQNIVLLLGLSWLVPLCCETLVISLS-STEGRKKFMQTCLPHLCCLFNVTASLLFDLMYSRY-HLRNFMA
Fr3OR6030.1             MSLERYVAVCYPLRHAALITIGNTGLMISVIWAFSLLSVLIQVSMV---TSSQKARNTLLLHLVQLGLSLSSTVYKPILAAL-RLQNVLY
Fr3OR633.1              MVVDTYAAVRWPLHYHDILPPARTHRILFGVWFLAAVYPFTLVVLM-WENRFSRARVTLLAHGLLLLLYFAPGFVFTLELFL-WVSTVNM
Fr3OR7011.1             MTLERYVAICLPLRHPELCSLHNTQKCILMILTVSSVPCFIILSTF---KSTWKGLSTVILHAFQLLLCLIQLWCPYIESAI-NVRYFNY
Fr3OR7149.1             MAYDRYVAICYPLQYSLIMNTERVCKIILGVWGYSVMNFLVLFSFF-SKENTQKAVSTCTPQIVSLSNMFLGTLFHFSESRI-KLRIILS
Gg2OR1.2                MAFDRYVAVCDPLRYASTLTPSVIGKMALTAVLRGFCIMFPPIFLL-SPETRLKTLSTCGSHLCVILMFYTPAFFSFLTHRF-HVHILLA
Gg2OR5.19               MAYDCYVAICSPLLYSTVMTKRVCMQLVVGSYMGGLLNSLTHTCGL-SDDGRKKTFHTCASHLTAITIFFGSLAFSYIQPSS-EQEKVSA
Gg2OR9.1                MVLDTYIAILFPLRYIAILPSSRTRKVIVLLWVCSGALPGVFFLVL-WESICSRASVTFLMHNTVFFFYFSPLLVLFVESFL-WISLTIC
Gg2ORUn.61              MAVDRYFAICHPLRHSTILSMPVVAALGSLVLLRGFLMVGPACFLL-ASDTQLKAFNTCVSHICVILAFYVSGLFTFLTHRF-HIHILVA
C.auratus OR1           LAYDRLIAICWPLRYSTINTNLRMLLIIAGIWSLVVLLEIFPVIFA-SISQRWKAFHTCLTHLLLVLLYYMPVILAYVLGNL-DLFTAVL
C.auratus OR2           MAFDRYVAICNPLRYPNIVTKLNIFCLCLAAWVLTNTCPLMMAIRA-STQGRLKSFSTCSPQLIIIALYFLPRCFIYLSSNI-DLRLVII
C.auratus OR3           MAYDRYVAICRPLEYHSVMTNQRIIECILFCWLSPFFCMCVVIVLT-SKEGRNKFIQTCVPHLLALLNVSFALLFDVLYTRY-DLRNFMA
L.fluviatilis_OR        MSVDRYLAIGYPLRYHSLMSCRTAIVAFMAATASCVLMCLGNVGVV-LRSGQKKALKTCGTHFLVVTVFFAAIFFSFASGLS-SLRYTLQ
B.belcheri OR           MAIDLYYFVCDPLHYHAKVTTKRVIIGIVTIRAFSLFFGFGLAPMA-LWVFQTKAFKKMAPHAIVLTVTVATAIFQVATVRV-VAENVVI
O.latipes OR1           MSFDRYVSICKPLQYQSLMSKRRVTVMLILSWLWPLFQLTVAISGK-SKEVRSKAAQTCLPHLLVLVNFSCLSSFDVIIGFF-TLHLTVM
O.latipes OR2           LCYDRLVAIMFPLHYQVRVTHKIMLSIVFFFWLFVITAVLITVGLL-TAHERVKALKTCTAHLSLVAIYFIPILITFTMRAN-NARIINL
Dr_OR108-1              LSYDRFIAICFPLRHESINTNTRMTYIICVSWFLSFSISLYGTLSI-NNQRRFKALATCTEHLILVSLYFFPIAIIFSLGFF-SVRTVCL
Dr_OR104-1              MALDRYLAICHPLRYPVLMTNRTMRLLVGFSWVTAMIAPSISLSLT-NAKGRLKALSTCATQLTIISIYYVPRFAVYITSNV-AEKIALV
Dr_OR106-1              MAIDRLIAIRLPLRYHSIVTNSRTFLFIVLTWVIAIALMGVLTSAV-NNSSRKQMINTCLSHLVVLLSYYAPKIVSALLTRI-TERNAIL
Dr_OR109-1              LAYDRLVAICFPLQYHAIVTKSAMFLIIGVMWIVSVTFFSILVGLV-HGAERIKALKTCTSHFMLVAMYYLPVLTNIIASAT-NVQIINN
```

```
D.rerio OR     MSLERYVAVCFPLRHCTIATPKRTGIGIGIIWFLSTINIITDIILA-DKDSAKKALKTVMLHLIQLGLCLTSFLYVTIERTL-NLRYLNY
Dr_OR115-1     MAFDRYVAICNPLRYTAIMTNKMVVKLSASAWGLSIFMVSILLGLT-NKSLNSKAIKTCSTHIAVYLIMFISCASFIFLHRV-DTRKLAS
Dr_OR101-1     MAFDRYVAICNPLRYTTIMTRKLCVLLIFASWTYGALIVLPPVIWA-SMAQKLKAGATCVSHLIVVFISYCCAFFVYISYRV-EVRIMIA
Dr_OR137-1     MAVERYIAVCKPLHHHQICTVRRTYILISLIWSVGVLPGLAELILL-NKSSAKKAQNTILLHGAQLLLCMLSYITPVLDRLY-RIRILNY
Dr_OR134-1     MAIERYIAICYPLRHAQICTVHRAYVLIGVIWFVSVVPDITDLFVT-ENTSAKRATNTILLHGLQLIMCLLSYISPSVEGIL-EIRFANY
Dr_OR128-2     MAFDRFAAICKPLRYHSIVRPRFLACLIVINLTFPMILLGVSALLT-SAQLKGKAYQTCIPHIVVLLNFTIAVTCDVTLSRV-GLSVFLS
Dr_OR121-1     MSFDRYVSICYPLQYNRIMTPVRVGLLIALVWIYCFIQFFIFLSFN-SKEAKKKALSTCTPHIISLLNFTLGWLFEILQSRF-LIRVIIS
Dr_OR113-1     MALDRYFAICKPLYYHKYMEMSNFLKFVFAPVIRNALLIVTMVSLA-SGTANMKALNTCVTHLIVLSISLTSALTAFLSYRI-NNRIFIS
Dr_OR118-1     MAYDRYITICCPLRYGAIMTTNNLLRIIIGMWLFNVVAITVICVLL-QSDARVKALQTCGTHLVVFLLLEINTLFPLLAHRS-FLRRVFS
Dr_OR111-1     MAYDRLIAICLPLRYHSIVNNSNMILIFSAIWAFNSSVVALMVSLI-TWEGRLKALKTCVSHLLLVGIFFLPLFCTYLAQLL-NARVIST
Dr_OR103-5     MAIDRYTAICYPLQYHSIMTNRNVLILILSPWILSFWGPLALVIRA-GSQGRMKTFSTCSPQLIIIALYFIPRILNFLSSNI-DLRLIMI
Dr_OR102-2     MALDRFIAICNPLRYHTLIKNSTILIICAFVWTANMLQLVGVTLLS-KSEGRYKTFSTCTPQLLIICLYYLPRTFVYITNIS-DTRMVVS
Dr_OR124-1     MAVDRYIAICRPLHYHRIMSSFTVLSLVTFIWVFSCFVGLMAISLT-SKVSREKFFSTCIPHLISFLNTTCGLSDSLYTKFN-VEHTFLS
Dr_OR125-1     MSYDRYVAICKPLDYHSRLTKNTCVKLILFSWIVPTSFSVASILLS-SLENRKKFWQTCLPHIFSLINFTFAFLFDFMYNRY-SLRHFLA
Dr_OR133-1     MSLERYVAICFPLRHAEITTYGRTNMAIGVIWMISWIQSLSEIVIF-DKASAKKATKTVLLHLIQLGLCAASILIGVIQEVI-SFMFFFF
Dr_OR112-1     MALDRFVAICKPLHYNEIMNSSMFLKLFLFTLIRSGTIMSTLVALA-AGEDRWKAFHTCGTHLMVMICFYFVGSVTFLSRNL-DVNTFLG
Dr_OR130-1     MCVERYVAICLPLRHHAISTSRRAFMVILMIWIVSSINPFIDMFIL--KKSASKGQRTISLHILQLFLCTAEVMCPYIESVV-TVRFINF
X.laevis OR1   MAVDRYVAICKPLRYHSIINNRLVTLICYFCLVCAALIGLAITLIA-NNGNWQKAFYTCTTHLIVIGLYFIPRLFVYSITQI-DINVLIL
X.laevis OR2   MAYDRYVAICYPLHYALRMSLKHCAKIIVGVWVAGFLDPVLHSVLI-SSEGKKKTLSTCTSHLTCVIIFYGTMMCLYLRPTK-PNQDIFA
X.laevis_OR3   MSYDRYVAICIPLRYTSIMSHRVCVTFILISWATGFGIAVLSVNPI-SNTGRQKAFSTCSSHLAVVSLFYGSIIAIYVVPPT-TINKVLS
Bf1OR44        MAIDIYYFICYPLQYETKVTTSRLAIGMVLVDTFSFICTSLPLILV-VKFYKTRGFKTMAPLAILLTTTIVSTLFMTVTVAQ-ILEKAST
Bf1OR45        MAMELYYFICQPLHYHQKVTTKRVAAGTVVVLALSLMLRVVYITIE-LWLYQTKAFKRLAPHAISLAVWVGTIVLLVVMTRQ-ITTRIAV
Bf1OR50        MAWDRYRAICDPFHYQEEDGIRWTGVRVVASWVWSMSLSAVSAIFI-GNPQGDRPRRTISIHLAIFIIFLTAVFTNAIMAIF-VLKRAAQ
Bf1OR52        MAMELYYFICQPLHYHQKVTTKRVAFGMVAVLVTSTLLRVVYITVE-LWLCQTSAFKNLAPHATSLAVWVGTIVLLVALSHP-LIRRIGI
Bf1OR53        MAIDLYYFVCDPLHYHDKVTTKRVVVGIVTIRAYSLLFGLVPVAFS-LWVFQTKAFKMMVPHVSVWSITIATTIFQAAMARA-VADDVSI
Bf1OR54        MAVDLYYFVCDPLHYHDKVTTKRVVVGIVMIRAFSLLFGLGPTAFS-LWIFQIKAFKMMVPQVGVWVISVATVIFRVAMARD-VAQHVSI
Bf1OR56        MATELYHFICNPLHYRSKVTTRRVIIGIVSVRAFALIFGIGQTLIK-LWIYQTKAFKALVPHIVVLVVSVASYVFLVASGRA-ITVIVAN
Bf1OR58        MAVELYYFICQPLHYNSKLTTKRVVFGILAVRAFALLFGAGSAIIQ-LWLCQTKAFKTLAPHIIVLAVQLASYIDMIALARV-ITSQVAK
Bf1OR59        MAVELYYFICQPLHYHGKVTTKRVVFGILAVRAFALLFGAGSAIIQ-LWLCQTKAFKTLAPHIIVLAVQLASFIFMIALARV-ITSQVAK
Bf1OR61        MAMELYYFICQPLHYHQKVTTKRVVVGMVGVLAISLLLRVAYIAVE-LWLYQTRAFKKLAPHVISLAVWGGTVVLMIVSSRQ-IITRIAA
Bf1OR12        MAWDRYQAICNALHYQMKGSLRCLCLKIAASWTFSALLGVVYVTRF-LRDVWKAYILTIATHVVPEDLNLLPGQLID------VRRTLEQ
Bf1OR37        MALELYYFICKPLHYRAKVTTKRVIIGIFAVRTFALVFGVGPELLE-LWLCQTKAFNVMAPHITVLAVSVASLIVMVVSIRA-ITVKVSK
Bf1OR46        MAWDRYQAICNALHYQTKGSLRCLCLKIAAFWTFSALLGVVYVTRF-LRDVWKAYILTIATHVVPEDLNLLPGQLID------VRRTLEQ
Bf1OR51        MALELYYFICKPLHYRAKVTTKRVIIGIFAVRTFALVFGVGPELLE-LWLYQTKAFNVMAPHITVLAVSVASLIVMVVSIRA-ITVKVSK
Bf1OR28        MAVELYYFICQPLHYNSKLTTKRVVFGILAVRAFALLFGAGSAIIQ-LWLCQTKAFKTLAPHIIVLAVQLASFIFVIALARV-ITSQVAK
Bf1OR60        MAVELYYFICQPLHYNSKLTTKRVVFGILAVRAFALLFGAGSAIIQ-LWLCQTKAFKTLAPHIIVLAVQLASYIVMIALARV-ITSQVAK
Bf1OR41        MAVDLYYFVCNPLHYHDKVTTKRVTVGIVTIRAFSFFFGLAPVAFG-LWVFQTKAFKMMAPQATVWAISVATVIFQVAIRVL-ILDHVSS
Bf1OR11        MAMDVYYFIIHPLHYNDRVTRTLTFAVALTSVAVGGSGSVQDVSDV-LQLHETQAFRALAPHFTVVLVFVGTAILSFIFARA-VARRVAM
Bf1OR1         IAADVYFFTIHPLHYNESITTKRLGVAVVVFRAISLCLSVVTVVVG-LSLRETRAFRTLAPSFAIVLVFVLANTMCFVTIRL-VARRISL
Bf1OR2         MAMDVYFFIIHPLHYNDKVTTPRVAWAIAAARTLTFVVALTSVAVG-LQLHETQAFRALAPHFTVVLVFVGTAILSFIFARA-VARRVAM
Bf1OR13        MAWDRYQAVCNALHYQEKLDLPRLLTKIAAAWTCSLALSLAYIISM-SDSSQRNKRTERTISAQVTILFVFLIIRYIQIGIS-IVKRTVQ
Bf1OR14        MAWDRYQAVCNALHYQEKLDLPRLLTKIAAAWTCSLALSLGYIISM-SDSSQRNKRTERTISAQVTILFVFLIIRYIQIGIS-IVKRTVQ
Bf1OR5         MAVDLYYFVCDPLHYHDKVTTKRVAVG----------IGLGPTAFS-LWIFQTKAFKMMAPHVSVWAISVATVIFRVAMARS-VADDVSI
Bf1OR38        MAVELYYFICKPLHYNGEVTRKRVTIGIAGVRVIAIIFGLGPMVAK-LWLYQTKAFKTIAPHFIVVMVSAATLAFVSATFRS-ITAHVAK
Bf1OR8         MAVDLYHFVCNPLHYHDKVTTKRVVVGILTIRAYSLFFGIASVASG-LWVFQTKAFKLMLPHLIVWTVSIATICFTVAMGRA-IADDVSI
Bf1OR9         MAVDLYHFMCNPLHYHDKVTTKWVVAGILTVRAYSLFVGIASVAFG-LLVFQTKAFKLMVTHAIVLTVSAVTVGFQVAIGRA-IAEHVSI
```

```
Bf1OR10               MATDLYHFVCNPLHYHDKVTTKRVVAGILTIRAYTLFFGIAFVAFD-LWVFQTKAFKLMLPHLMVWTVAAVTVGFQVAMKQA-IANQMST
Bf1OR4                MAVDLYHFVCNPLHYHDKVTTKRVVAGILTIRAYSLFFGIASVAFG-LWVFQTKAFKLMLPHVIVWTVLLATVSYNIAMHME-IANHVSI
Bf1OR19               MAIDLYYFVCDPLHYHDKVTTKRVVVGIVMIRAFSFLFGLGPTAFS-LWIFQIKAFKMMVPQAGVWVISVATVIFRVALSQN-VAEHVSI
Bf1OR15               MGLDLYYFVCDPLHYHDKVTTKRVVVGILVIRAYSLLFGLGPMAFS-LWVFQTKAFKMMMPHATVWTISVATVFFQTAMARA-IADHVSI
Bf1OR26               MAMELYYFICQPLHYHQKVTTKRVAFGMVAVLVTSTLLRVVYITVE-LWLCQTSAFKKLALHAISLAVWVGTVVLLVALSRP-LVRRIGI
Bf1OR30               MAVDLYYFVCDPLHYHDKVTTKRVAVGILVIRAFSLFFGLAPVAFG-LWVFQTKAFKLMAPHAIVMTLSLATAIFQLAMARA-VANHVSV
Bf1OR18               MAVDLYYFVCDPLHYHDKVTTKRVVVGIVAIRAFSLFFGLGPLAIG-LWVFQTKAFKLMAPQAIVMTVSVTTTVFQIAMARA-VANHVSI
Bf1OR27               MAVELYYFICQPLHYHGKVTTKRVAFGILAVRAFALLFGAGSAIIQ-LWLCQTKAFKTLAPHIIVLAVQLVSYIVMIALARV-ITSQVAK
Bf1OR24               MAIDLYQFVCHPLHYHERVTTKRVGVSILTIRAFSLFLVAAPVAFG-LWVFQTKAFKMMLPHAIVLTVSVATTVFQVAITRA-VADHVSA
Bf1OR23               MAIDLYYFVCDPLHYHDKVTTKRVALGIVTIRAFSLFFGLGPVAFS-LWIFQTKAFKTMVPHAIVWIVFVSTMILLVAMGRA-IANHVAN
Bf1OR29               MAVDLYYFVCDPLRYHDKVTTKRVAVGIVVIRAFSLFFGLAPVAFG-LWVFQTKAFKLIAPHTIILTVSIATTVVQIAMARA-IADHVSI
Bf1OR22               MAWDRYQAICNPFHYQEVDAVHWTGIRVLATWVWSISLSVVSVIFA-GGGDNHSPRRTISIHLSIFMIFMTTVCANGVIGHM-VLKRVVQ
Bf1OR40               MAWDRYQAICNPFHYQEVEAVRWTGIRVLAAWVWSISLSVVSVIFV-GGGDNHSPRRTISIHLSIFMIFMTTVCANGVIGHM-VLKRVVQ
Bf1OR25               MAWDRYRAICDPFHYHDEDGIRWTGIRILVCWVLSTVTRVLCVVCT-GGGDSHRPWRTISIHLAILVIFSATCLLNGVIHAV-VLKRVIQ
Bf1OR31               MAIDMYYFICHPLRYNTQVSVRKVALGMLVVDVLAISIGITPVAIA-LRIYQTRAFKTMGGHVIILLVFLSATIFLIVSRRA-IAHKVAV
Bf1OR36               MAWDRYRAICDPFHYHDEDGIRWTGIRILAAWVFATATSVLCVVCT-GGGDSHRPWRTISIHLAILVIFSATGLLNGVIHAV-VLKRVIQ
Bf1OR3                MAVDLYYFVCDPLHYHDKVTTKRVAVGIVTVRAFSFFFGLAPVAFG-LWVFQTKAFKLMAPHAIALTVSVTTTVFQVAMARA-VAHHLSI
Bf1OR32               MAVDLYYFVCDPLHYHDKVTTKRVAVGIVAVRAFSFFFGLGPVAFG-LWVFQTKAFKLMAPHAIALTVSVTTTVFQVAMARA-VAHHLSI
Bf1OR34               MAVELYYFICKPLHYNGEVTTKSVTIGIAGVRVIAIIFGLGPMVAK-LWLYQTKAFKTIAPHFIVVMVSATTLAFVSATFRS-ITAQVAK
Bf1OR17               MGLDLYYFVCDPLHYHDKVTTKRVIVGIFATRTYSLLFGLGPLAFS-GLPKKGLPCEIDP----------------------ANSVSFI
Bf1OR39               MAVDLYYLVCEPLHYHDKVTIKRVVVGIATIRTLSLLFGLAPVVFF-LWVFQTKAFKMMVPHAIVLTVSVATSIFQVAMARA-VADHVAI
Bf1OR6                MAMDRYYVICWALNYHNVVTTSRVLSLITAAWLTSIVSVAVYQIMS-VKFLETKAVKTITTHSIIFLIFFVSWSLHGITKLL-AANRTFL
Bf1OR21               MAIDIYYFICYPLQYETKVTTSRLAIGMVLVDTFSFICTSLPLILV-VKFYKTRGFKTMAPLAILLTTTIVSTLFMTVTVAQ-ILEKASI
Bf1OR43               MAVDLYHFVCNPLHYHDKVTTKRVVAGIVTIRALSLFFGLAPVAFG-LWVFQTKAFKMMAPQATVWAISVATVIFQVAIRVL-ILDPVSS
H.sapiens OR2L5       MAYDRYVAICFPLHYPIRMSKRMYVLMITGSWMIGSINSCAHTVYA-SAEGRKKAYSTCSTHLTVVTFYYAPFAYTYLCPRS-TEDKVLA
M.musculus Olfr683    MAYDRYVAICKPLQYPSIITDQFVVRAAIFVAARNGILTMPIPILS-AEGAVAKALSTCGSHFILILFFSTVLLVLVITNLA-DVPILLN
Xt1OR10411.1          MSIDRYVAICNPLQYSAIMRKELSVELVTLCWFLGYSFAFNLTVLI-STTGRQKALSTCSSHLAVVATFLGSLIGLYLLPSS-TANKILS
Xt1OR11576.2          MAYDTYWAVCHPLHYAAIMTNRKILRLVLASLFMNCLLVGTAVLLS-SENAGRKAAHTLVTHLLNFSIFLVATLFIFIRYRL-IVHVILS
Xt1OR2286.1           MALDRYLAICRPLRYHNIMNNRLVGQLFLIGLVQSSLFSSSIIIAV-RGKALHKTLHTCSTHLIVVVLNYSCGLSSSILYRM-DVQNLFS
Xt1OR35679.1          MAIDRYVAICHPLRYHSIISNKLATLLCYFLWPFAALNGLAMTLIS-NNENWQKAFYTCTTHLLVIGLYFIPRLFLHVINQY-DANVLIV
Xt1OR39249.1          MAYDRYVCICIPLRYNSLMSLYTVLRLIAAAWVYAVVQFTVQLVLT-SSTHRSKALQTCTPHMMSLTYFVLDLISEALLNRS-ELRVLIS
Xt1OR42821.1          MAFDRYVAICNPLRYKVILSNGTIIRTSALAVIRGVICILPLFLLA-PSTAGLKAFGTCVSHVCAILTFYVPILVSSLVHRF-PTHILLA
Xt1OR5329.1           LSLERYIAISHPLRHKELCTERRCNAAMVGMWVVGLIPVIADFITM-RSSSAFSAGKTVLLHAFQLLLCMFSFTYTVTETYL-FLPVINF
Xt1OR5508.1           MIVNVCIAVCWPLKYLAFVHAVKFKIIACIWIIAFFEPFLSVLYEI-FNRSSQQAKRTILIHALQIVLHFLPTLVTIWVGGK-IIFDLAN
M.musculus Taar7a     ISVDRYIAVSDPLTYPTRFTASVSGKCITFSWLLSIIYSFSLLYTG-VAKRERKAAKTLGIAVAAFLLSWLPYFIDSIIDAF-TYVYEIL
R.norvegicus Taar5    ISIDRHCAICDPLLYPSKFTVRIALRYIAAGWGIPAAYTAFFLYTD-AVKRERKAAKTLGIAVGIYLVCWLPFTVDTLVDSL-PLVFDIF
H.sapiens Taar2       VAIDRFYAICYPLLYSTKITIPVIKRLLLLCWSVPGAFAFGVVFSE-QVKKDKKAAKTLGIVIGVFLLCWFPCFFTILLDPF-VVLFDAL
R.norvegicus Taar3    IAIDRFYAVCAPLHYTTTMTASMIKRLLFFCWAAPALFSFGLVLSE-SKKKDRKAAKTLGIVMGVFLACWLPCFLAVLIDPY-IIVLDLL
M.musculus Taar4      ISVDRHYAVCDPLHYVTQITTRVVGVFLLISWSVPIFFAFGLVFSE-TSKKESKATKTLSIVMGVFVLCWLPFFVLTITDPF-EDLYNVF
D.rerio Taar66        IAVDRHQAVCFPLQYPTMITIPVAWVMVIISWSMAAFYSYGLVYSK-SRRSERKAAKTLGIVVGAFVICWLPFFINSMMDPY-GVLFEAF
D.rerio Taar1b        VSAERFCAVCGPLRYRSCFGLSTVLLMISISWLIPGIFAYVMTFLE-VQLQTRKATVTIAIVVGAFLVCWTPFFLCNILNPF-PMLIDVL
H.sapiens P2Y1 recep  ISAHRYSGVVYPLKSLGRLKKKNAICISVLVWLIVVVAISPILFYS-NSPLRRKSIYLVIIVLTVFAVSYIPFHVMKTMNLR-YATYQVT
H.sapiens P2Y11 rece  ISLNRYLGIVHPFFARSHLRPKHAWAVSAAGWVLAALLAMPTLSFS-GTADHGLAAYRAYSLVLAGLGCGLPLLLTLAAYGA-YVGYQVM
H.sapiens P2Y12 rece  ITIDRYQKTTRPFKTSNPKNLLGAKILSVVIWAFMFLLSLPNMILT-GKVPRKKVNVKVFIIIAVFFICFVPFHFARIPYTL-FYVKEST
D.rerio P2Y receptor  ISVHRYLGICHPIRSLTLIKPRHAHMVCGFVWTAVIACLVPTLILV-QSSSRKKSIKLIIVVLVVFAICFVPFHITRTLYYA-NFSYKIT
H.sapiens hypocretin  IALDRWYAICHPLMFKSTAKRARNSIVIIWIVSCIIMIPQAIVMEC-QIRARRKTARMLMIVLLVFAICYLPISILNVLKRV-YAWFTFS
```

```
D.rerio hypocretin r  IAQDRWYAICHPLKFKSTAKRARKSIVLIWLVSCIMMIPQAVVMES-QVKARRKTARMLMVVLFVFALCYLPISILNIMKRV-YAWFTFS
H.sapiens oxytocin r  MSLDRCLAICQPLRSLRRRTDRLAVLATWLGCLVASAPQVHIFSLR-ISKAKIRTVKMTFIIVLAFIVCWTPFFFVQMWSVW-ASAFIIV
G.gallus oxytocin re  MSLDRCLAICQPLRSLHRRADRVSVLLTWLLCLLVSIPQIHIFSLR-ISKAKIRTVKMTFIIVLAFIVCWTPFFFVQMWSVW-ASPFIIA
H.sapiens opioid rec  MSVDRYIAVCHPVKALDFRTPAKAKLINICIWVLASGVGVPIMVMA-KDRSLRRITRMVLVVVGAFVVCWAPIHIFVIVWTL-VAALHLC
D.rerio opioid recep  MSVDRYVAVCHPVKALDFRTPMKAKIINILIWVLSSAAGIPAMVLG-KDRNLRRITRLVLVVVAVFVVCWTPIHIFILVKAL-MAAYFFC
H.sapiens somatostat  MSVDRYLAVVHPLSSARWRRPRVAKLASAAAWVLSLCMSLPLLVFA-RRRSERKVTRMVLVVVLVFAGCWLPFFTVNIVNLA-AGLYFFV
D.rerio somatostatin  MSIDRYMAVVHPIRSARWRRPSVAKVINSMVWALSCLLTLPVIIYC-RRKSEKKVTRMVVIIVVVFVICWLPFFMLNIFNLV-TGVYFLT
D.rerio alpha2B-adre  ISLDRYMSISRAVTYGPKRTPKRIKCAILVVWLISAVISFPPLLSM-MVNREKRFTFVLAVVIGVFVICWFPFFFSYSLQAV-EPLFKFF
R.norvegicus alpha2B  ISLDRYWAVSRALEYNSKRTPRRIKCIILTVWLIAAVISLPPLIYK-QLSREKRFTFVLAVVIGVFVVCWFPFFFSYSLGAI-HGLFQFF
D.rerio adenosine re  IAVDRYIAIKIPLRYNSLVTGRRAKGIIAVCWILSVVIGLTPMFGW-TLQKEVHAAKSLAIIVGLFAVCWLPLHIINCFTLF-DWVMYLA
B.taurus adenosine A  IAVDRYLRVKIPLRYKTVVTPRRAVVAITGCWILSFVVGLTPMFGW-YYGKELKIAKSLALILFLFALSWLPLHILNCITLF-RILIYIA
H.sapiens melanocort  IAVDRYVTIFYALRYHSIMTVRKALTLIVAIWVCCGVCGVVFIVYS-QQHSCMKGAVTITILLGVFIFCWAPFFLHLVLIIT-TAHFNTY
D.rerio melanocortin  IAADRYITIFYALRYHSIMTTQRAVGIILVVWLASITSSSLFIVYH-RQTTSMKGAITLTILLGVFILCWGPFFLHLILILT-FSHFNLF
B.floridae 225039     LTAERYWFIVHGMTYVSNVTNDKCKVVVVIVWMLSVALAMLPNFGW-ESSTSRRSAITVGIVTVAFLVGWLPLLIKMSFPSD---VSDVH
B.floridae 227418     IALDRYWAITDAVKYMHQRTTRRAVVMVALVWVASLCISVPPLFGW-VFSKERRAAKTLGIITGVFIVCWLPFFLVALIEPF-PLGRSVI
B.floridae 107702     LSINSYVGVRHPIYFHIHANSAKLRAVVAMIVSWIIFSLIAFSPSM-KYEARVYRSRTVMIYVVVAFVFWLVPLLLMAVCSR-WIGPEIL
B.floridae 202803     IAYDRYKAIVFPTEPRMSLGKMRNALAGIWIGAVAVMVPQVFVLHV-VSKKRVRVLKMLITVVVLFALSWLPLYTCWMLGDF-QYVYPVA
B.floridae 211803     IAVDRFYSVVHPTEPKITIRGVQKIVIAVWLLALFIMVPQVLLIED-SWKRKVKVVKMLLAVVVLFAFSWLPLHTLSLLSDH-VYIYPIA
B.floridae 148901     MSIDRYLAISHPFRSMSFRTRKVATFTNIGVWVASLASISPVLAFA-AEKKTKKVAKMVLVVVVVFLVCWLPYYVIALVNMQ-LVSYFVS
B.floridae 92625      MSVDRYMAIVHPVDSIDVRKPGLAWAVELVIWLLAMGASAPQLVYF---RAKRKVTKMLIVVVVVFGMCWLPHHILNMWVIF-YAFNLLA
M.musculus mas proto  ISVERCLSVLYPIWYTSHRPKHQSAFVCALLCALSCLVTTMEYVMC-WASHSSKLYIVIMVTIIIFLIFAMPMRVLYLLYYE-GNLHNIS
H.sapiens mas-relate  ISTERCLSILWPIWYHCRRPRYLSSVMCVLLWALSLLRSILEWMFC-RKMPLTRLYVTILLTVLVFLLCGLPFGIQWALFSR-CHVHLVS
H.sapiens Fpr2        IALDRCICVLHPVWAQNHRTVSLAMKVIVGPWILALVLTLPVFLFL-GMIKSSRPLRVLTAVVASFFICWFPFQLVALLGTV-DILVNPT
M.musculus Fpr-rs3    IAMDRCTCVLHPVWVQNHRTVSLARKVIVGAWILSLLLTLPHFLFL-GFLNSSRPLRVLTAVAISFFMCWFPFQLIILLGNI-HILLNPA
M.musculus Fpr-rs2    IALDRCICVLHPVWAQNHRTVSLARKVVVGPWIFALILTLPIFIFL-NLVNSSRPLRVLTAVVASFFICWFPFQLVALLGTV-DMFVNPT
M.musculus Fpr1       IALDRCICVLHPVWAQNHRTVSLAKKVIIVPWICAFLLTLPVIIRL-GLIKSSRPLRVLSFVVAAFFLCWCPFQVVALISTI-VTALKIT
D.rerio Fpr-like      ISVDRALCVWCPVFTRKRRTVCAARMVSVGVWIMAGIFSLPYFVYR-RLSGKSRPLRILAVLVCAFFLCWAPYHFLRLVRFV-KLGWRMV

                              190       200
                      ....|....|....|....|...
Dr3OR10.21            SLTQTIPPMLNPILYTLKTDEVM
Dr3OR10.24            SLRQVIPSMLNPIIYTLKTEEVM
Dr3OR10.8             IAASLVPPLINPTVYCTRTKEIR
Dr3OR15.35            SLSYAVPPLLNPIIYVFNTAEIK
Dr3OR15.41            SLAYTISPMLNPIIYVLNTAEIK
Dr3OR17.1             LYFLVCPPLFNPIMYGVRLANIK
Dr3OR21.16            LELVIVSPVFNPLIYGLNVRAIR
Dr3OR21.21            IVFLIFPKCLCPLIYGMRDQAFS
Fr3OR142.7            MMYSLIPAAINPLIYCFKTKDIK
Fr3OR18.1             FAFFSLAQCIAPVVYGLRKEELQ
Fr3OR2346.3           IQFLIITPVLNPIIYGLKLTNIR
Fr3OR6030.1           VCLFILPRCLSALIYGIRDQLIR
Fr3OR633.1            CVFMLLPRAFAPYLYGLRYREIS
Fr3OR7011.1           VTFILAPRCLSPLIYGLRDEKFL
Fr3OR7149.1           IYLLLIQPMITPFLYGFNLPKIR
Gg2OR1.2              NLYVVVPPMLNPIVYGVRTRQIR
```

Gg2OR5.19	VFYTLLVPMLNPLIYSLRNKEVR
Gg2OR9.1	NVLMILPKVLFPFLYGLRYREIS
Gg2ORUn.61	DIYLLLPPTLNPIIYGVRTKQLR
C.auratus OR1	TVSVTLPPMLNPIIYSLKTDELR
C.auratus OR2	MMYSLFPPMINPLIYCLKTKDVK
C.auratus OR3	LEFLLVPPMLNPLIYGLNLTKLR
L.fluviatilis_OR	TAQYVFPPALNPVIYGLRTAEIR
B.belcheri OR	LMFLTLSSMANPIIYSFRLPEFR
O.latipes OR1	LQIIIYQPLFNPIIYGLKMKEIS
O.latipes OR2	SLTSVFPPMLNPIIYVLQTQEIR
Dr_OR108-1	SLSSVLPPCANPVIYSLKTKEIR
Dr_OR104-1	MFYSLLPPLMNPFIYFIRIREIR
Dr_OR106-1	IISSLIPPLINPTVYCTRTKEIR
Dr_OR109-1	SLTQAIPPMLNPIIYTLKTEEVM
D.rerio OR	IIVLILPRCLSPLIYGMRDEAVW
Dr_OR115-1	IMFHIVPPGLNPLVYGFQTKEIR
Dr_OR101-1	LLYSVLTPLLNPIIYSLRNKELQ
Dr_OR137-1	LLTNILPRLLTPLIYGVRDKQFY
Dr_OR134-1	LIVYILPRFLSPIIYGVRDKKFR
Dr_OR128-2	LEFLIIPPVLNPLIYAFNLPDIR
Dr_OR121-1	VYFLMCQPLLNPILYGVRLKNIR
Dr_OR113-1	TVYLFFPSCLHPLIYGWRTKEIR
Dr_OR118-1	ISNFIFPPLVNPLIYGFKTKEIR
Dr_OR111-1	SLSYAIPPMLNPIIYVLNTGEIK
Dr_OR103-5	MMYSVLPPLINPIIYCLRTEEVK
Dr_OR102-2	MMYSLIPAVINPFIYCFRTKEIK
Dr_OR124-1	IIILTVPPFLNPLIYGIKLGPIR
Dr_OR125-1	LELVIVPPLFNPLMYGLNIRAVR
Dr_OR133-1	VVFMIFPRCLSPLIYGLRDQAFS
Dr_OR112-1	VMYIVFPASVNPIIYGVRTKEIR
Dr_OR130-1	LAFSITSRAVSPLVYGFRDEKFY
X.laevis OR1	CLYTFIPHIASPIIFCLGTKEIR
X.laevis OR2	LLYVVLIPMLNPFIYTLKNTEFK
X.laevis_OR3	LMFTLVIPMTNPIIYSLKSTDIR
Bf1OR44	VLG-----SASP-----------
Bf1OR45	LLYTTLSSMVNPIVYSFRMPDFR
Bf1OR50	LVYLTVPCFTNPLIYGFRSEELR
Bf1OR52	LLYTTLSSMVNPIVYSFRMPDFR
Bf1OR53	LLFLTVSSIANPIIYSFRLRDFR
Bf1OR54	LLFLTVSSVANPIIYSFRLREFR
Bf1OR56	RIFLTLSSMVNPIVYSFRRPEFR
Bf1OR58	LIYLTLSSVVNPIIYGFRHPEVR
Bf1OR59	LIYLTLSSVVNPIIYGFRHPEVR
Bf1OR61	LLYTTLSSMVNPIVYSFRMPDFR
Bf1OR12	ETYRLASPPPPVPPHCGKVTRSA
Bf1OR37	LLYQTVSSMVNPIVYSFRQPEFR

Bf1OR46	ETYRLASPPPPVPPHCGKVTRSA
Bf1OR51	MLYQTVSSMVNPIVYSFKQPEFR
Bf1OR28	LIYLTLSSVVNPIIYGFRHPEVQ
Bf1OR60	LIYLTLSSVVNPIIYGFRHPEVR
Bf1OR41	LLYLTVSSVANPIIYSFRLPDFR
Bf1OR11	LLNLSLSYITDSVVYSLKKADFR
Bf1OR1	LLGLTLSSVTDAVFYSLNQTHFR
Bf1OR2	LLNLSLSYITDSVVYSLKKADFR
Bf1OR13	FLYLTVPCFSNPVIYGLSSKELQ
Bf1OR14	FLYLTVPCFSNPVIYGLSSKELQ
Bf1OR5	LLFLTVSSIANPIIYSFRLREFR
Bf1OR38	LLNITVSSMVNPIVYSFCRPEFR
Bf1OR8	LLFLTLSSIANPILYSFRLPEFR
Bf1OR9	LLYLTLSSMANPIIYSFRLPKFR
Bf1OR10	LLYLTVSSMANPILYSFRLPEFR
Bf1OR4	LLYLTVSSMANPILYSFRLPEFR
Bf1OR19	LLFLTVSSIANPIIYSFRLREFR
Bf1OR15	LMYLTVSSMANPIVYSLRLQEFR
Bf1OR26	LLYTTLSSMVNPIIYSFRMPDFR
Bf1OR30	LLFLSVSSVANPIIYSLRLPDFR
Bf1OR18	LLFLTVSSVANPIIYSFRLPDFR
Bf1OR27	LIYLTLSSVVNPIIYGFRHPEVR
Bf1OR24	LLFLTLSSMANPIICSFRLPEFR
Bf1OR23	ILFLTLSSMANPIIYSFRLPEFR
Bf1OR29	LLFLTVSSVANPIIYSLRLPDFR
Bf1OR22	LVYLTVPCFSNPMIYGFRSEEIR
Bf1OR40	LVYLTVPCFSNPMIYGFRSEEIR
Bf1OR25	LVYLTVPSFANPIIYGFRSEEIR
Bf1OR31	HLFRTLSPMADPLVHSLRVPDFR
Bf1OR36	LVYMTVPSFTNPIIYGFRSEEIR
Bf1OR3	LLFLTVSSVANPIIYSFRLPDFR
Bf1OR32	LLFLTVSSVANPIIYSFRLPDFR
Bf1OR34	LLNITVSSMVNPIVYSFCRPEFR
Bf1OR17	LMFLTVSSMANPIVYSFRLREFR
Bf1OR39	LLFLTVSSIANPIIYSFRLPDFR
Bf1OR6	LVFLTIPCYSNPIVYGFCNTEIQ
Bf1OR21	LLNLTLSSMLNPIIYSLRLPEFR
Bf1OR43	LLYLTVSSMANPIIYSFRLPDFR
H.sapiens OR2L5	VFYTILTPMLNPIIYSLRNKEVM
M.musculus Olfr683	ILHHLIPPALNPIVYGVRTREIK
Xt1OR10411.1	LLYTVVTPLFNPIIYSLRNWEIR
Xt1OR11576.2	ASGLIFPPLLNPLIYGVRTKALK
Xt1OR2286.1	AIYYLFPATIHPIIYGYRMKEIR
Xt1OR35679.1	CLYTFIPHLASPIIFCLRTKEIR
Xt1OR39249.1	VQVFVIAPFLNPLIYGLKLKEIR
Xt1OR42821.1	NFYLLIPPMLNPLVYGMKTKKIQ

Xt1OR5329.1	FFFMCLPRFISPMIYGMRDEVFR
Xt1OR5508.1	FIIFSFAQCLSPVVYGLRCTELR
M.musculus Taar7a	VWIAYYNSAMNPLIYAFFYPWFR
R.norvegicus Taar5	IWFAYFNSACNPIIYVFSYRWFR
H.sapiens Taar2	TWFGYFNSTCNPLIYGFFYPWFR
R.norvegicus Taar3	VWLGYFNSTCNPLIHGFFYPWFR
M.musculus Taar4	LWLGYFNSTFNPIIYGMFYPWFR
D.rerio Taar66	VWLGYMNSAINPIIYGLFYPWFR
D.rerio Taar1b	MWFGYANSTLNPFIYAFMHSWCR
H.sapiens P2Y1 recep	RGLASLNSCVDPILYFLAGDTFR
H.sapiens P2Y11 rece	RGLMPLAFCVHPLLYMAAVPSLG
H.sapiens P2Y12 rece	LWLTSLNACLDPFIYFFLCKSFR
D.rerio P2Y receptor	RPLASVNSCLDPILYFLAGDHYR
H.sapiens hypocretin	HWLVYANSAANPIIYNFLSGKFR
D.rerio hypocretin r	HWLIYANSAANPIIYNFLSGKFR
H.sapiens oxytocin r	MLLASLNSCCNPWIYMLFTGHLF
G.gallus oxytocin re	MLLASLNSCCNPWIYMLYTGHLF
H.sapiens opioid rec	IALGYANSSLNPVLYAFLDENFK
D.rerio opioid recep	VALGYTNSSLNPILYAFLDENFK
H.sapiens somatostat	VILSYANSCANPVLYGFLSDNFR
D.rerio somatostatin	VILTYVNSCANPLLYGFLSDNFK
D.rerio alpha2B-adre	FWIGYCNSCLNPVIYTIFNKDFR
R.norvegicus alpha2B	FWIGYCNSSLNPVIYTVFNQDFR
D.rerio adenosine re	IILSHANSVVNPFIYAYRIRDFR
B.taurus adenosine A	IFLSHGNSAMNPIVYAFRIQKFR
H.sapiens melanocort	LVLIMCNSVIDPLIYAFRSLELR
D.rerio melanocortin	LILIICNSLIDPLIYAYRSQELR
B.floridae 225039	MVFMILNSAMNPVIYGFRLSEVR
B.floridae 227418	TWLGYANSAMNPVLYGFVNKDFQ
B.floridae 107702	FSFYSLNSIINPMATLIRTEELR
B.floridae 202803	HWLGYSNSCANPIVYGFFNTNIR
B.floridae 211803	HWLSYFNSSVNPIIYGYYNENFR
B.floridae 148901	ICLGYANSCINPFIYLSFSAKFR
B.floridae 92625	MCLAYVNSCVNPIIYAFLSSEFR
M.musculus mas proto	LLFSTINSSANPFIYFFVGSSKK
H.sapiens mas-relate	IFLSALNSSANPIIYFFVGSFRQ
H.sapiens Fpr2	SSLAFFNSCLNPMLYVFVGQDFR
M.musculus Fpr-rs3	STLASFNSCLNPILYVFLGQEFR
M.musculus Fpr-rs2	SSLAYFNSCLNPMLYVFMGQDFR
M.musculus Fpr1	SPLAFFNSCLNPMLYVFMGQDFR
D.rerio Fpr-like	SDLTYFNSCINPVLYLFMGLDVR
