# Supplementary figures and images for: Amphioxus (Branchiostoma floridae) has orthologs of vertebrate odorant receptors
Source: BMC Evol Biol. 2009 Oct 5;9:242. doi: 10.1186/1471-2148-9-242 (PMC2764704; doi:10.1186/1471-2148-9-242)

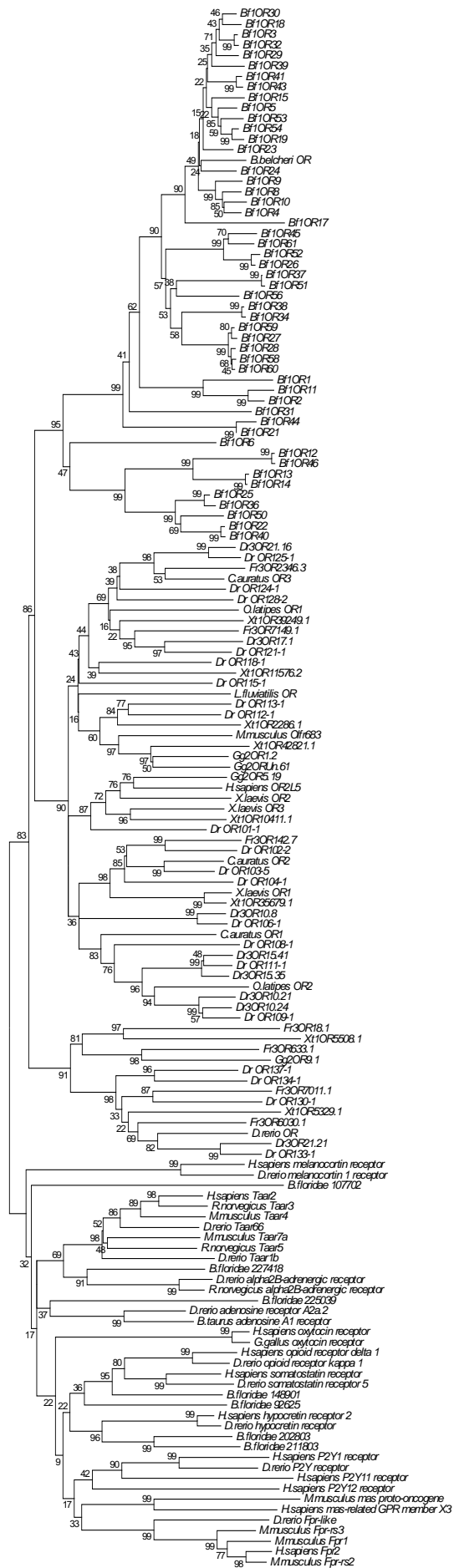

Supplement: Additional file 4 — Phylogenetic analysis of vertebrate ORs, B. floridae ORs and non-OR Rhodopsin-like GPCRs. This file contains an unrooted Neighbor-Joining tree constructed using vertebrate ORs, cephalochordate ORs and non-OR GPCRs from the α, β, γ and δ groups of GPCRs from the Rhodopsin family [20]. Non-OR GPCRs include melanocortin, trace amine-associated, alpha2B-adrenergic, adenosine, oxytocin, opioid, somatostatin, hypocretin, mas-related, formyl peptide and purinergic receptors. Fish-mammalian pairs of receptors were included where possible as well as non-OR B. floridae GPCRs identified by [16] (see Additional file 2 for the complete sequence list). Tree construction was based on approximately 200 amino acid positions and 1000 bootstrap replicates were conducted (see Additional file 3 for sequence alignment). [file 1471-2148-9-242-S4.PDF]
